# Optimal Schedules of Light Exposure for Rapidly Correcting Circadian Misalignment

**DOI:** 10.1371/journal.pcbi.1003523

**Published:** 2014-04-10

**Authors:** Kirill Serkh, Daniel B. Forger

**Affiliations:** 1 Department of Applied Mathematics, Graduate School of Arts & Sciences, Yale University, New Haven, Connecticut, United States of America; 2 Department of Mathematics, University of Michigan, Ann Arbor, Michigan, United States of America; 3 Computational Medicine and Bioinformatics, University of Michigan, Ann Arbor, Michigan, United States of America; Indiana University, United States of America

## Abstract

Jet lag arises from a misalignment of circadian biological timing with the timing of human activity, and is caused by rapid transmeridian travel. Jet lag's symptoms, such as depressed cognitive alertness, also arise from work and social schedules misaligned with the timing of the circadian clock. Using experimentally validated mathematical models, we develop a new methodology to find mathematically optimal schedules of light exposure and avoidance for rapidly re-entraining the human circadian system. In simulations, our schedules are found to significantly outperform other recently proposed schedules. Moreover, our schedules appear to be significantly more robust to both noise in light and to inter-individual variations in endogenous circadian period than other proposed schedules. By comparing the optimal schedules for thousands of different situations, and by using general mathematical arguments, we are also able to translate our findings into general principles of optimal circadian re-entrainment. These principles include: 1) a class of schedules where circadian amplitude is only slightly perturbed, optimal for dim light and for small shifts 2) another class of schedules where shifting occurs along the shortest path in phase-space, optimal for bright light and for large shifts 3) the determination that short light pulses are less effective than sustained light if the goal is to re-entrain quickly, and 4) the determination that length of daytime should be significantly shorter when delaying the clock than when advancing it.

## Author Summary

When our body's internal timekeeping system becomes misaligned with the time of day in the outside world, many negative effects can be felt, including decreased performance, improper sleep, and jet lag. When misalignment is prolonged, it can also lead to serious medical conditions, including cancer, cardiovascular disease, and possibly even late-onset diabetes. Rapid readjustment of our internal daily (circadian) clock by properly timed exposure to light, which is the strongest signal to our internal circadian clock, is therefore important to the large proportion of the population which suffers from misalignment, including transmeridian travelers, shift workers, and individuals with circadian disorders. Here we develop a methodology to determine schedules of light exposure which may shift the human circadian clock in the minimum time. By calculating thousands of schedules, we show how the human circadian pacemaker is predicted to be capable of shifting much more rapidly than previously thought, simply by adjusting the timing of the beginning and end of each day. Schedules are summarized into general principles of optimal shifting, which can be applied without knowledge of the schedules themselves.

## Introduction

Modern society requires individuals to be awake and alert at times that conflict with their internal circadian (∼24-hour) timekeeping systems. In year 2012 over 60 million Americans traveled overseas, subjecting themselves to long periods of circadian mistiming, impaired sleep, and low performance [1]. Over one fifth of American workers follow non-standard schedules, which place them at increased risk for sleep-related accidents [2], [3]. Circadian misalignment has also been linked to many health problems [4], [5]. Thus, after a schedule shift, it is important for individuals to reach a state of proper circadian timing (entrainment) as quickly as possible, and to minimize the time spent between entrained states (re-entrainment).

Light is the strongest signal to the human circadian system [6]. Light can slow (phase delay), or speed (phase advance) the circadian clock, depending on when it is administered. When properly timed, light can reduce the amplitude of the circadian clock, making it more sensitive to subsequent light signals [7]. Previous light history has been shown to affect re-entrainment as well, and may also increase or decrease sensitivity to light [8]. A large literature exists offering suggestions on how to time light exposure to quicken re-entrainment and avoid jet lag. Suggestions include additional pulses of light [9], amplitude suppression [10], intermittent light [11], [12], and avoidance of morning light [13]. These suggestions are important, since it has been shown that the choice of light schedule can significantly quicken [14] or slow [15] re-entrainment. Given the infinite variety of possible schedules, however, it is unrealistic to attempt to find schedules that re-entrain in the minimum amount of time through experimental methods alone.

Accurate mathematical models for the effect of light on the human circadian system are available [16], [17]. Several studies have demonstrated their value in practical applications. Specifically they have been used to design experimental protocols i.e. the so-called “forced desynchony” protocol [18]; their predictions have been validated against experimental data [19]; and they have been used effectively in numerous real-world applications [20]. These models contain two components: “Process L,” which simulates phototransduction in the retina [21], and a “Process P,” whose two variables represent the state of the central circadian pacemaker, located in the suprachiasmatic nucleus of the hypothalamus. The choice of a “Process P” model with at least two variables is important, because it represents both circadian phase and amplitude [22]. More detailed biochemical models are available [23], but these can be reduced to models similar to [16] and [17], and have yet to be fit to human data, making them a less attractive choice. Additionally, many features of the biochemical models, such as a narrow range of entrainment [24] or the presence of a sharp threshold separating orthodromic (the same direction as the schedule shift) and antidromic (the opposite direction) re-entrainment [25], are captured by models of this type. The process of re-entrainment involves multiple oscillators (i.e. in tissues of the body [26] and in regions of the SCN [27]), the dynamics of which are not captured by a single-oscillator model. Multiple-oscillator models are available i.e. [28], however while such models are very promising for studying re-entrainment, they too have not yet been fit to human PRC data and are far less widely used than [16] and [17]. This makes them less attractive, at least until their parameters are fit to human data.

Mathematical models can, in theory, be analyzed to determine optimal schedules, or schedules which outperform all others [29]. In practice, however, this analysis is quite difficult. For this reason, previous studies have used mathematical simplifications, which unfortunately severely restrict the types of schedules considered. These prior studies have light exposures of a fixed intensity and duration [9], ignore the dynamics of phototransduction [30], [31], optimize each time-point separately [30], [32], [33], rather than considering how to optimize the whole schedule together, minimize light levels, rather than minimizing time to entrainment [34], [35], and consider only schedules where circadian amplitude is relatively unperturbed [9]. The problem with these prior studies is that such simplifications have been shown to yield suboptimal schedules, which can result in nearly double the amount of time needed to re-entrain when compared with optimal schedules [31].

Here we describe a mathematically robust method, which uses existing mathematical models [16], [17], without any simplifying assumptions, to produce schedules that are locally optimal. These schedules are proven to outperform any other schedules which are not locally optimal. (A detailed statement of these methods is provided in supplemental text S1) We also show how these schedules and this methodology can be applied to the more subtle problem of partial re-entrainment (See Designing Schedules for Partial Re-entrainment in supplemental text S1). Our method is fast, accurate, and broadly applicable to a wide variety of problems of biological oscillation. It avoids the difficulties encountered in prior studies by accomplishing the following: 1) It requires as a constraint that the final phase be exactly entrained, but allows, using a penalty, other variables (including circadian amplitude) to deviate from their average values within experimentally observed ranges. 2) It solves some equations forward in time and other equations backwards in time. 3) It recognizes that the best light schedules are **bang-bang** (terms which are bold/underlined are defined in the glossary, supplemental text S2), and consist of periods of either darkness or maximum light levels, and 4) It uses the **slam shift** as a starting point for the optimization. This last part of our methodology is not an assumption, but a property of optimal schedules. We justify this usage in the Methods section.

Using this approach, we determined over 1,000 schedules that optimally re-entrain, without the limits on the length of schedules imposed by prior studies. Moreover, while previous work often assumed that the light available for shifting was 10,000 lux or higher, we considered many light levels, including those found indoors.

## Results

Each optimal schedule for *complete* re-entrainment gives the pattern of light and dark (LD) which will entrain the existing model [16] in the minimum time (See supplemental text S1). Each optimal schedule for *partial* re-entrainment gives the LD pattern which will place CBTmin at the start of the SD region in the minimum time (See Designing Schedules for Partial Re-entrainment in supplemental text S1). Optimal schedules for partial re-entrainment can be derived from those for complete re-entrainment. Both types of schedules consist of only two light levels—one as dim as possible and the other as bright as possible. This is not an assumption, rather, we demonstrate that light should always be at its minimum or maximum level if the goal is to entrain quickly. Moreover, each 24-hour phase of the schedule consists of one day phase and one night phase. Thus to shift optimally, a traveler needs only to change the timing of his or her dawn and dusk. This is both practical and somewhat surprising, since we find these optimal schedules to outperform many others, including schedules which are extremely difficult to follow (i.e. those with continuously fluctuating light levels, or with multiple light/dark phases i.e. an LDLD cycle). It is also encouraging that the optimality of long light exposures agrees with previous studies, which have asserted that while brief pulses of light may be much more effective per photon of light, continuous light still provides the most drive to the circadian system [21].

**Figure 1:**
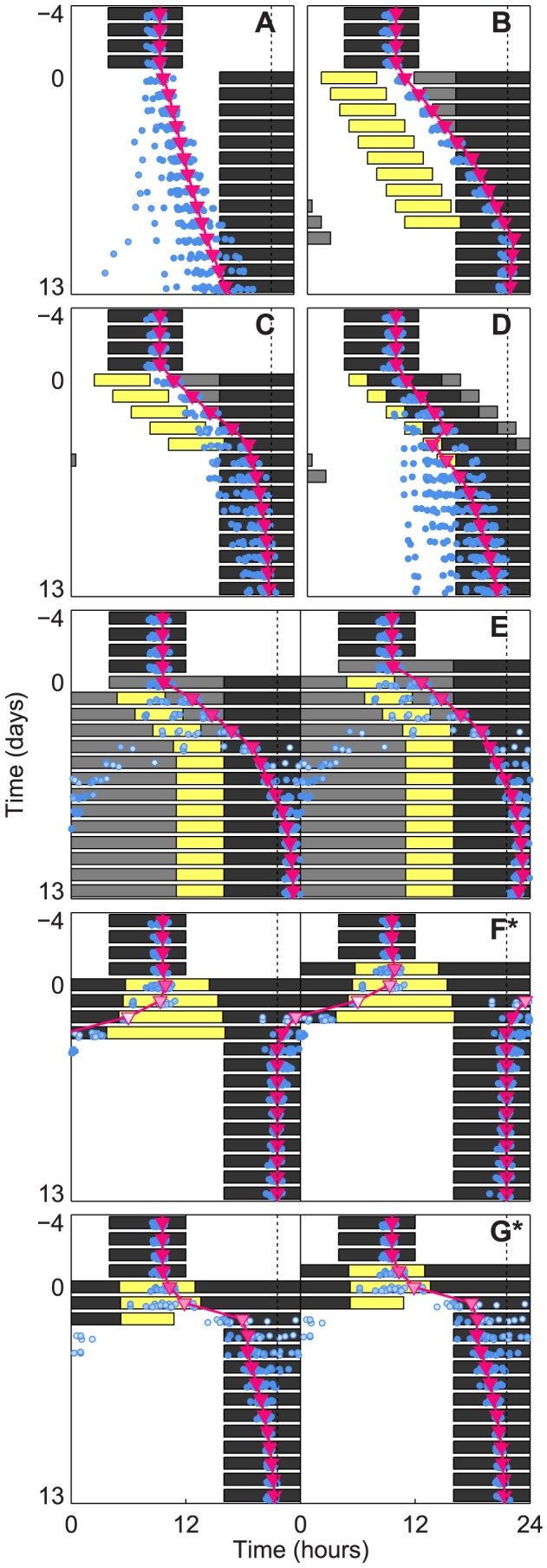
Comparison of schedules for a 12-hour shift of the light-dark cycle. Predicted circadian phase, indicated by simulated core body temperature minima (CBTmin, magenta triangles), is plotted against the pattern of exposure to bright light (10,000 lux, yellow), moderate light (100 lux, white), dim light (5 lux, gray), and darkness (0 lux, black). Predicted CBTmin under noisy light levels (See supplemental figure S1), with circadian period randomly sampled from an experimentally measured distribution [18], is plotted for 20 hypothetical subjects (blue circles). Circadian amplitude at CBTmin is indicated by the brightness of the markers, with white corresponding to zero amplitude. The timing of entrained CBTmin in the new time zone is indicated by the dotted line. The subjects are initially entrained to a 16∶8 LD-cycle in moderate light. At day 0 the schedule shift occurs. The six schedules are compared are: (**A**) The abruptly shifted LD-cycle; also called a slam shift. (**B**) Times of light exposure and avoidance in the new time zone are prescribed to quicken re-entrainment. Phase delays of 1 hour/day are assumed. Based on the recommendations proposed by R. Sack (See Supplementary Appendix of [49].) (**C**) Times of light exposure and avoidance are prescribed, with an assumed phase delay of 2 hours/day. Based on the recommendations proposed by J. Waterhouse et.al. (See Table 2 of [50].) (**D**) The sleep/dark region is gradually delayed, with 2 hours of bright light before bed and 2 hours of light avoidance after wake. Assumed delay of 2 hours/day. Based on the recommendations proposed by C. Eastman et.al. in [51]. (**E**) A PRC is used to place a series of 5 hour light stimuli, in a background of dim light, in order to produce a large delay. The timings are refined using a model [16]. Proposed by D. Dean et.al. (See Figure S1:E1 in [9].) (**F**) Our optimal schedule for complete re-entrainment in minimum time. A model [16] is used to compute the mathematically optimal schedule of light exposure (See Methods) which resets the model in the least possible amount of time. (**G**) Our optimal schedule for partial re-entrainment in minimum time, designed to place CBTmin at the beginning of the sleep/dark region as quickly as possible (See Designing schedules for Partial Reentrainment in supplemental text S1).

In figure 1, we compare our optimal schedules (1F and 1G) to five other previously proposed schedules (1A–1E) for re-entrainment to a 12-hour time-zone shift (1A). We present all seven schedules as actograms, where each new line represents a subsequent day. Black indicates darkness. Gray indicates dim light (5 lux). White indicates low room light (100 lux). Yellow indicates bright light (10,000 lux). In the original time zone, days −4 to −1 show a light-dark schedule of 16 hours of 100 lux light and 8 hours of darkness (LD 16∶8). At time 0 of day 0, we assume a transition occurs. A magenta triangle shows the predicted timing of the core body temperature minimum (CBTmin), a key circadian marker that, when entrained, occurs slightly after the midpoint of the dark episode. The brightness of the triangle's face represents the strength of the timekeeping signal, or the **circadian amplitude**, with white corresponding to zero amplitude. A blue dot also shows the predicted timing of CBTmin, except under conditions approximating real-world variations in light-levels and inter-individual differences, the details of which are explained in the sequel. The blue dots predict the CBTmin of twenty hypothetical subjects, rather than one.

**Figure 2:**
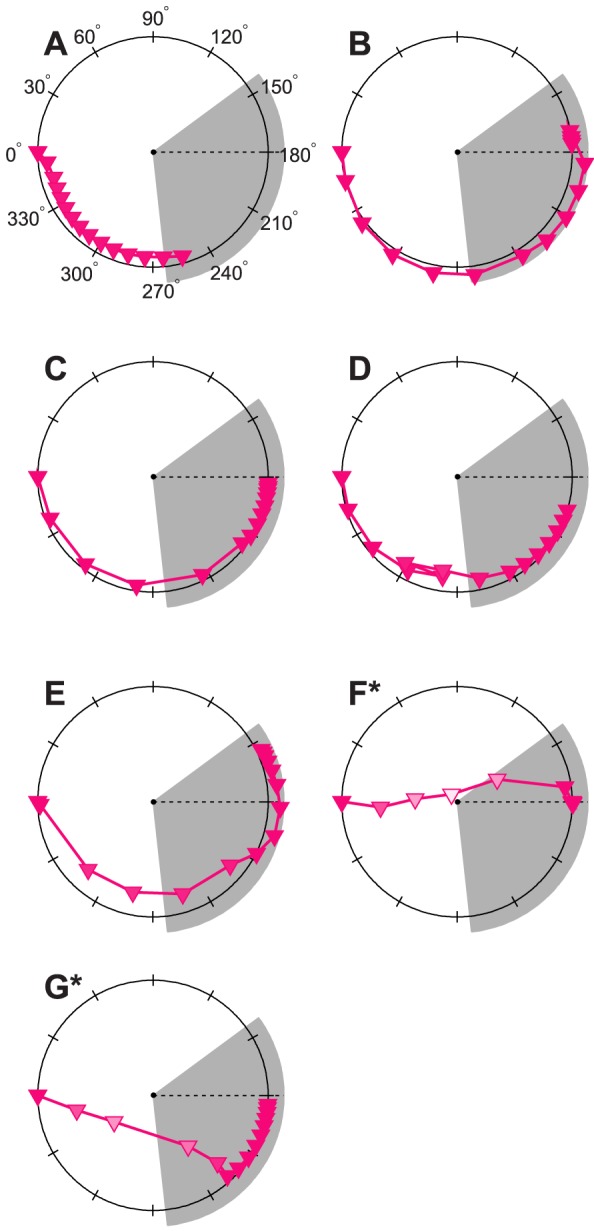
Polar phase-amplitude resetting maps corresponding to figure 1. Circadian phase is plotted in degrees, with 0° corresponding to entrained CBTmin (before the schedule shift occurs), while amplitude is measured on the radius. A 6 hour advance would be indicated by a shift to 90°, a 12 hour shift to 180°, and a 6 hour delay to 270°. The sleep/dark region is indicated by the shaded regions on the PARMs. The timing of entrained CBTmin in the new time zone is indicated by the dotted line. Figures (**A**)–(**G**) correspond directly to figures 1A–1G, with each subplot displaying the process re-entrainment under one of the six schedules considered in figure 1. The phase and amplitude of predicted CBTmin (magenta triangles) are plotted in a one-to-one correspondence with the markers of figure 1. In other words, the markers and lines of figure 1 are simply re-plotted here in phase-amplitude space. As in figure 1, re-entrainment is plotted for 4 days before the schedule shift and for 14 days after.

To study the effects of these schedules, we also plot the process of re-entrainment in terms of both phase and amplitude. Thus, we produce experimentally measurable predictions for phase-amplitude resetting maps (PARMs) [36] in figure 2. PARMs plot phase and amplitude simultaneously – the angle to the origin corresponds to the phase, and the radius corresponds to the limit-cycle amplitude. These maps show clearly how the optimal schedules – both for complete re-entrainment (1F) and partial re-entrainment (1G) – shift the circadian pacemaker along the shortest and straightest paths in phase-space (the space of phase-amplitude pairs) (See figures 2F and 2G). The optimal schedules involve partial circadian amplitude suppression, which occurs when light exposure is centered near the crossover between advancing and delaying regions of the day [37], and can also be observed between advancing and delaying schedules [38]. The existence of this mode of re-entrainment, and of circadian amplitude suppression in general, has been shown experimentally [7], [36]. Moreover, amplitude suppression has been found to be consistent across multiple circadian variables [39].

Our proposed schedule for optimal (minimum-time) complete re-entrainment to a 12-hour time zone shift takes approximately 4 days, whereas the five previously proposed schedules require more than 7 (see Table 1). Moreover, several previously proposed schedules require more than 13 days to achieve complete re-entrainment. Our schedule for optimal partial re-entrainment takes only 2 days, while other previously proposed schedules take 3 days or more. These large gaps in predicted performance between optimal schedules and those previously suggested strongly imply that current methods have large room for improvement.

**Table 1:** Times to re-entrainment under the schedules presented in figure 1.

Schedule	Time to complete re-entrainment (days)	Time to partial re-entrainment (days)
Slam shift	>13	13
Sack et al. [49]	9	4
Waterhouse et al. [50]	13	3
Eastman et al. [51]	>13	6
Dean et al. [9]	7	3
Optimal schedule (complete)	4	3
Optimal schedule (partial)	13	2

The number of days required to achieve re-entrainment (both complete and partial) are recorded for each of the seven schedules presented in figure 1. The number of days does not include the first day on which re-entrainment occurs (i.e. if re-entrainment occurs on the third day then the number of days required is two). This way if the subject starts out re-entrained the number of days required is zero. Complete re-entrainment is said to occur when CBTmin falls within approximately one hour of the exactly entrained time (the dotted line in figure 1). Partial re-entrainment is said to occur when CBTmin falls within the sleep/dark region of the destination time zone.

Since real-world light levels are highly variable, we added variability in the lighting conditions to mimic the natural environment (See supplemental figure S1) [40]. We also allowed the period of the circadian pacemaker to be drawn at random from a distribution matching the human population [18]. We then re-simulated each schedule twenty times and plotted the predicted CBTmins with blue dots (figure 1). With the exception of figure 1B, which took more than ten days to achieve complete re-entrainment, we found that the other four schedules (1A, 1C, 1D, and 1E) were highly variable, while our optimal schedule (1F) had very little variability. Moreover, our optimal schedule for partial re-entrainment (1G) had most of its variability confined to the SD region, which still allows for good sleep. This is remarkable, since the optimal schedules we propose re-entrain in the minimum time, and are therefore not specifically optimized for robustness or noise. Thus we see that robustness is a *feature* of optimal, rapidly-shifting schedules.

**Figure 3:**
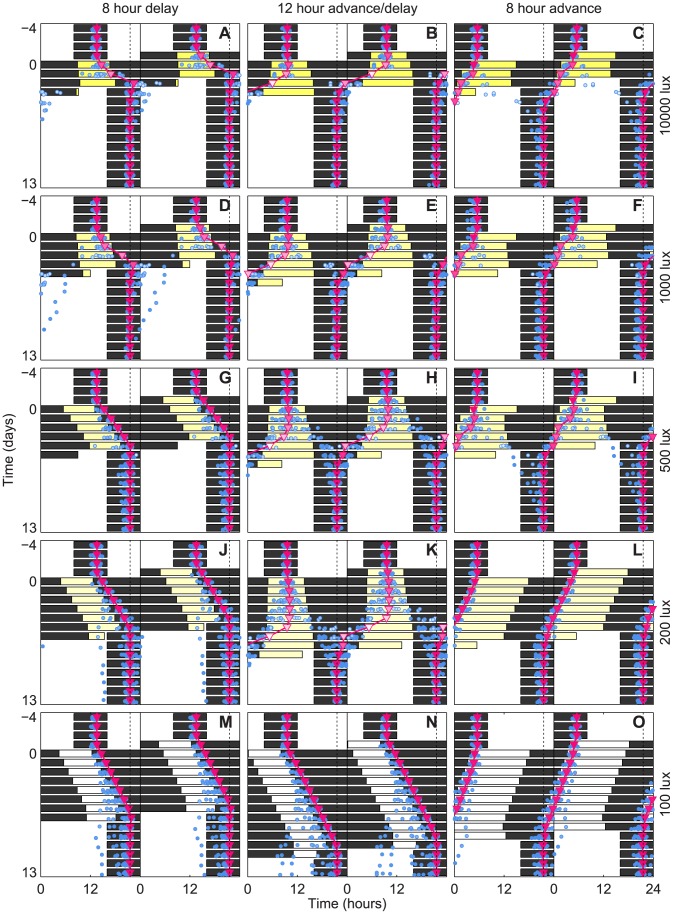
Optimal schedules for re-entrainment to 8 and 12 hour shifts of the LD-cycle. Predicted core body temperature minima (CBTmin, magenta triangles) are plotted against the pattern of optimal exposure to bright light (200 lux–10,000 lux, yellow), moderate light (100 lux, white), and darkness (0 lux, black). Predicted CBTmin under noisy light levels (See supplemental figure S1), with circadian period randomly sampled from an experimentally measured distribution [18], is plotted for 20 hypothetical subjects (blue circles). The timing of entrained CBTmin in the new time zone is indicated by the dotted line. Circadian amplitude at CBTmin is indicated by the brightness of the markers, with white corresponding to zero amplitude. The subjects are initially entrained to a 16∶8 LD-cycle in moderate light. At day 0 the schedule shift occurs. Optimal schedules are grouped in rows by maximum admissible bright light level (yellow), and in columns by effected phase shift. Figures (**A**), (**B**), (**C**) use a maximum light level of 10,000 lux; (**D**), (**E**), (**F**) use 1000 lux; (**G**), (**H**), (**I**) use 500 lux; (**J**), (**K**), (**L**) use 200 lux; (**M**), (**N**), (**O**) use 100 lux. Figures (**A**), (**D**), (**G**), (**J**), (**M**) are optimal schedules for an 8-hour delay; (**B**), (**E**), (**H**), (**K**), (**N**) a 12-hour shift; (**C**), (**F**), (**I**), (**L**), (**O**) an 8-hour advance.

We next computed optimal schedules for complete re-entrainment in minimum time to an 8 hour advance and an 8 hour delay (See figure 3). Again, re-entrainment was possible within three to four days when 10,000 lux of light was available, and our schedules were more reliable than those previously proposed (e.g. the so-called “slam shift”, in which the light/dark cycle is suddenly shifted to the new time zone). We did find antidromic re-entrainment (delaying when advancing should occur, a experimentally observed phenomenon [41]) in a small fraction of the simulated population when advancing by 8 hours (See 3C). We also found that to delay was easier than to advance (re-entrainment in two versus three days), matching conventional wisdom. The 8 hour advancing and delaying schedules also required less amplitude suppression than the 12 hour phase shifting schedule.

Reducing the maximum available light level ten-fold to 1,000 lux yielded optimal schedules similar to the schedules for 10,000 lux, except that complete re-entrainment required an additional day. Reducing the maximum light level an additional two-fold to 500 lux added another day to each schedule. While the daily light exposure in schedules for the 12 hour shift and 8 hour advance changed little with reduced light, the 8 hour delay showed very little amplitude suppression (3G) compared to when more light was available (3A and 3D). Reducing the maximal light level to 200 lux required an additional two to three days to shift. At this lower light level, no amplitude suppression occurred for the 8 hour advance or the 8 hour delay. When the maximum available light was further reduced to 100 lux, no amplitude suppression occurred for any shift. These transitions are clearly visible in the corresponding PARMs (See supplemental figure S2).

**Figure 4:**
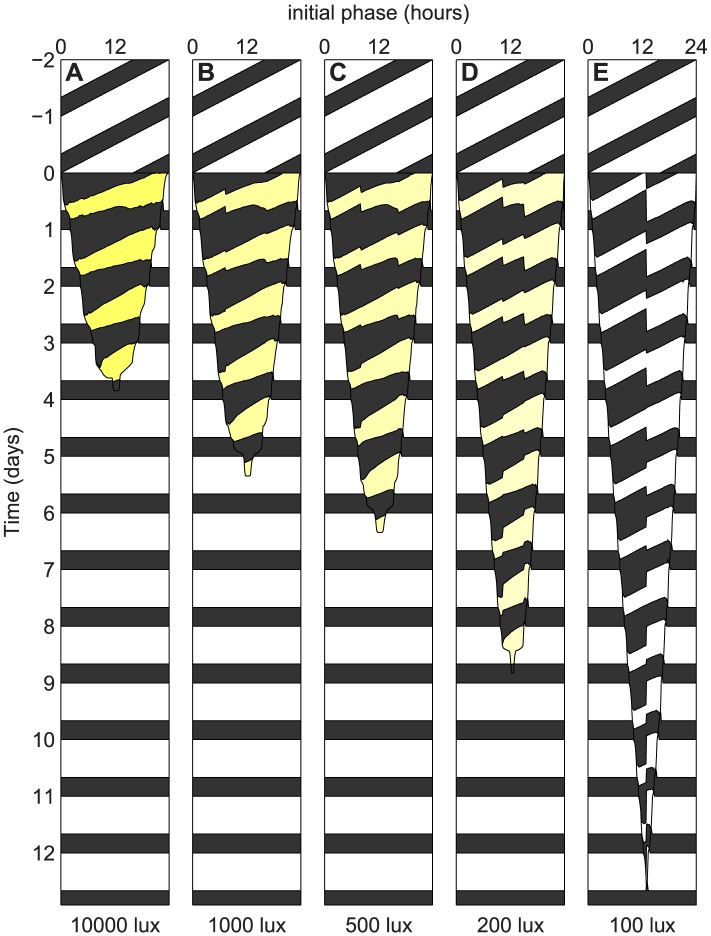
Optimal schedules for all phase shifts. This plot shows the pattern of bright light (200 lux–10,000 lux, yellow), background light (100 lux, white), and darkness (0 lux, black) under which the clock is optimally reset from the corresponding initial phase. If a vertical line is drawn on the plot, then the pattern of light and dark along this vertical line is the optimal schedule for resetting the clock from the corresponding initial phase. Figure (**A**) shows the optimal schedules for resetting the clock when 10,000 lux light is available; (**B**) 1000 lux; (**C**) 500 lux; (**D**) 200 lux; (**E**) 100 lux.

We next found optimal schedules for complete re-entrainment to all phase shifts with maximum light levels of 10,000, 1,000, 500, 200 and 100 lux consisting of approximately 1,000 optimized schedules. These are summarized in figure 4. Since schedules for partial re-entrainment in minimum time can be derived from schedules for complete re-entrainment (See Designing Schedules for Partial Re-entrainment in supplemental text S1), figure 4 can be used to find all optimal schedules for both complete and partial re-entrainment in minimum time.

The format used in figure 4 was described by Winfree in section 1D of [22]. It is interpreted in the following way. Suppose we were to draw a vertical line on figure 4A at initial phase 8. Each point on this vertical line would correspond to a time on the vertical axis. If we were to follow this line down from day −2 to day 13, we would find that this line passes through a white region (100 lux) for 8 hours, then a black region (0 lux) for 8 hours, then white for 16, black for 8, white for 8. Then it crosses time 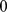, and passes through black for 8.9 hours, yellow (10,000 lux) for 7.8, black for 16.5, yellow for 8.1, and so on. This pattern of light and dark is the optimal schedule for re-entrainment to an 8-hour delay in 10,000 lux light – it corresponds exactly to days −2 to 13 of figure 3A (the optimal schedule itself only occupies days 0 to 3.5). Similarly, if we were to draw a vertical line on figure 4A at initial phase 12 hours and read off the schedule in the same way, we would get the optimal schedule for a 12-hour shift (figure 3B). A vertical line on figure 4A at initial phase 16 would likewise give the optimal schedule for an 8-hour advance (figure 3C).

**Figure 5:**
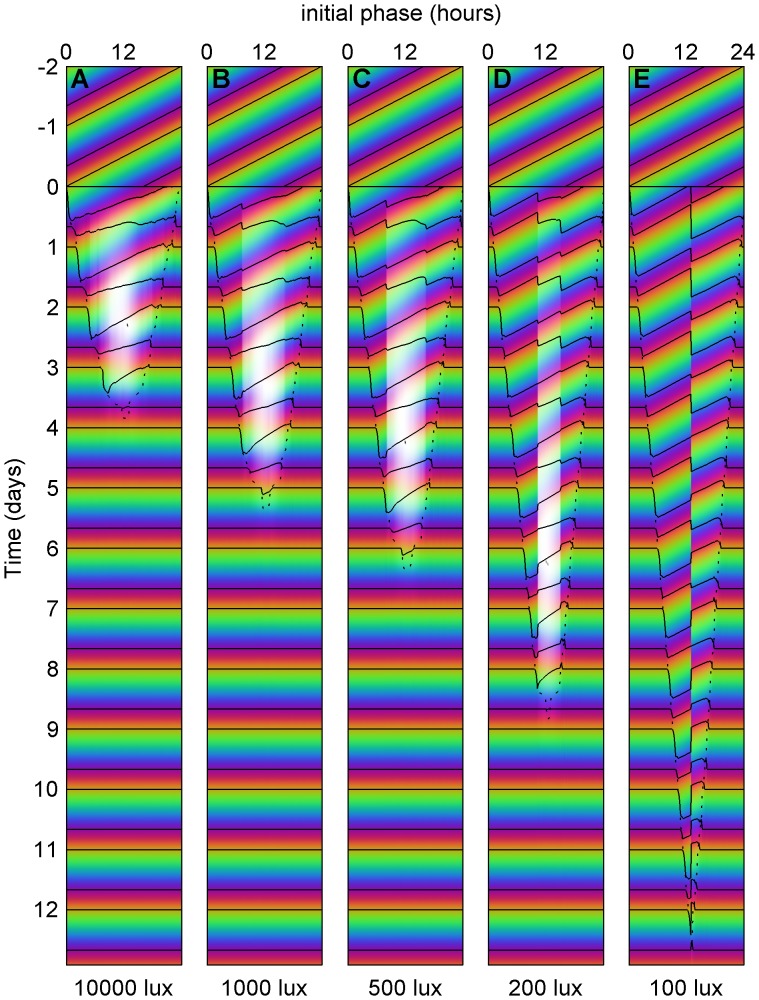
Predicted optimal circadian phase and amplitude for all phase shifts. The format is exactly the same as figure 4. Days −2 and −1 may be used as a legend associating a unique hue to each phase of the oscillator. Brightness is then used to represent amplitude, with white corresponding to zero amplitude [37]. The exact coloring, based on the state of the model variables, is shown in supplemental figure S3. Figure (**A**) shows the predicted phase and amplitude under the optimal schedules for resetting the clock when 10,000 lux light is available; (**B**) 1000 lux; (**C**) 500 lux; (**D**) 200 lux; (**E**) 100 lux.

The meaning of “initial phase” measured on the horizontal axis comes from the following interpretation. Without loss of generality, we fix our destination time zone, and consider shifts from all possible time zones. Each vertical slice corresponds to an optimal schedule for complete re-entrainment from the initial phase (where the slice intersects the horizontal axis) to phase zero. Days −2 and −1 show a 16∶8 light-dark (LD) cycle of 100 lux (dim home or office lighting) in the original time zone. At the beginning of day 0, the transition to the new time-zone begins. An optimal schedule for re-entrainment is presented, then once it is finished, the LD cycle in the target time zone takes over. Again, here black corresponds to darkness, white to dim light (100 lux), and yellow to bright light (200–10,000 lux depending on the intensity). We also simulate the predicted circadian response to these schedules, displaying both circadian phase and amplitude in figure 5. The format is identical to figure 4. Days −2 and −1 show the entrained state and thus may be used as a legend, associating a unique hue to each phase of the oscillator. After the transition occurs at day 0, the predicted state of the circadian pacemaker is shown, using hue to represent phase and brightness to represent amplitude. (The precise coloring, based on the state of the model variables, is shown in supplemental figure S3.) These schedules can be compared with the phase shifting predicted for a slam shift (See supplemental figure S4) which, particularly for lower light levels, does not phase shift the clock in the 12 days reported. Thus our optimal schedules are much more efficient than the slam shift.

From this plot (figure 5) and the PARMs (supplemental figure S2), we see that schedules can be separated into two classes. First, for 10,000 lux of light, or large (>8 hour) phase shifts with 1000 or 500 lux, the optimal schedules pursue the most direct path between the original state of the model and the re-entrained state. Thus in the PARMs the optimal schedule forms a nearly straight line between the state of the clock before the shift and the state after the shift. We call this pattern of optimal shifting minimum path shifting (MPS). For larger phase shifts with MPS (e.g. 8–12 hour time zone changes), the amplitude of the circadian pacemaker can be partially reduced in the middle of the schedule.

**Figure 6:**
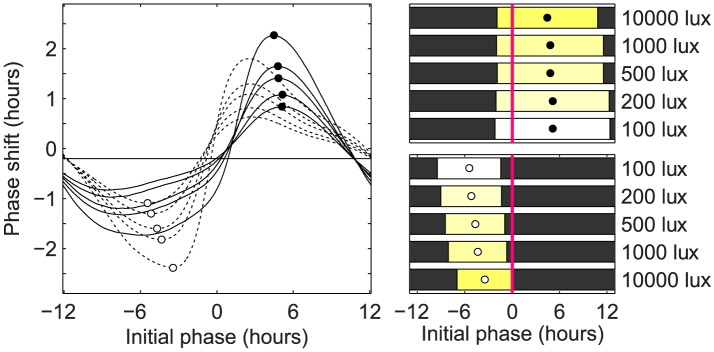
Optimal one-pulse stimuli to advance or delay the clock. We simulated the PRCs to all possible one-pulse stimuli for a variety of different light levels. For each light level, two stimuli were selected: the one producing the greatest advance and the one producing the greatest delay. The model was kept in total darkness before the stimulus was administered. Resulting phase shifts were measured using the concept of isochrons [22]. On the right the optimal advancing stimuli (top) and optimal delaying stimuli (bottom) are plotted for each light level. The bars indicate both the duration (bar length) and phase (midpoint) of the light stimuli relative to the timing of CBTmin (solid magenta vertical line). On the left the PRCs corresponding to each optimal stimulus length are plotted. On each PRC, the optimal phase maximizing the shift is marked by a circle (filled for advances and unfilled for delays). The PRCs corresponding to optimal advancing stimuli are drawn using solid lines, and ones corresponding to optimal delaying stimuli, using dashed lines. We found that, for low light levels and smaller shifts, the daily light exposures observed in the optimal schedules (See figures 4 and 5) matched the optimal one-pulse stimuli very closely. In particular, we find that the optimal advancing stimuli are much longer than the optimal delaying stimuli.

A second class of schedules is seen when the maximal light levels are lower (e.g. 200 or 100 lux) or for smaller (<8 hour) phase shifts with 500 lux or 1000 lux. These optimal schedules often shift with minimal changes to the preferred circadian amplitude. We call this type of shifting limit-cycle shifting (LCS). In LCS, light stimuli are presented which maximally advance or delay the circadian clock while near (but not on) the limit cycle; the next day's light stimulus is the same as the previous day's except that is presented at the appropriate time considering the phase shift predicted to occur. This is supported by figure 6: we compute the **phase response curves** (PRCs) to every possible 24-hour light pulse, and find that the stimuli which cause the maximum advances or delays in a single day match the optimal LCS schedules almost exactly. This is also supported by figure 4E (100 lux), in which no amplitude suppression occurs and therefore only LCS schedules are present. In this figure, the envelope indicating the time to re-entrainment is almost exactly a triangle – in the sense that the time to re-entrainment increases linearly with the magnitude of the phase shift. Moreover, from the fact that its peak occurs at a phase of 13 hours and a time of 12.7 days, we can estimate that the optimal number of hours delayed per day by 100 lux light is 13/12.7 = 1.03 hours/day and the optimal hours advanced is 11/12.7 = 0.87 hours/day. Compare this to figure 6, where the optimal number of hours delayed by a single stimulus of 100 lux light is 1.09 hours/day and the optimal hours advanced is 0.84 hours/day.

An important feature of LCS schedules is that the daily stimulus to delay the clock is much shorter than the stimulus to advance the clock. Moreover, there appears to be region of phases where no light appears in either stimulus. This goes against the predictions given by **instantaneous phase response curves** (iPRCs), which provide a simpler, amplitude-free model of circadian response [42]. This discrepancy arises from the fact that even in LCS schedules, a small amount of amplitude suppression still occurs. To validate the prediction that light pulses which maximally delay the circadian clock are significantly shorter than ones which maximally advance it, we look again to figure 6. We find that shorter pulses do in fact accentuate the delay regions of the PRCs, while longer pulses accentuate the advance regions.

Repeating these computations on the model described in [17] gave us almost the same results (See supplemental figures S5, S6, S7, S8, S9, S10, S11), the major difference being that the optimal schedules for the Simpler model [17] favor less amplitude suppression than for the Jewett-Forger-Kronauer model [16]. This is reasonable, since the dynamics of amplitude suppression and amplitude recovery differ greatly between the two models [43]. Specifically, in the equations for the oscillator, the Jewett-Forger-Kronauer model uses a seventh-order nonlinearity while the Simpler model uses only a cubic nonlinearity. The fact that two models which are both qualitatively and mathematically different gave similar answers suggests that we have found general principles of optimal shifting that aren't dependent on a specific model.

Finally, while we present the schedules in a form (i.e. figure 3) which could be applied to practical problems, there are several practical limitations which must be taken into consideration. Many of the theoretical schedules we propose require either very long sleep/dark phases or very long bright light phases, which may be difficult to reconcile with real-world obligations. This makes the use of brief pulses of bright light much more appealing [44], however as we have observed (e.g. figure 1) restricting ourselves to such a strategy may significantly prolong the time to re-entrain. One possibility is the use of low-transmission or red-blocking glasses [44] in conjunction with the use of a light visor [45]. While such a strategy may make our schedules much more practical, its effectiveness awaits experimental validation.

## Discussion

In this study, we have found locally optimal schedules which completely re-entrain the human circadian pacemaker in minimum time. The methodology we propose can determine the optimal schedules directly from the model, without any additional assumptions, and can create schedules which outperform any which are not locally optimal. Schedules are efficient, easy to follow, and robust to changing light levels and inter-individual differences. Our schedules are based on not one but two widely used mathematical models: [16] and [17]. The two models yielded very similar results. Schedules were summarized into general predictions including two classes of shifting, MPS and LCS.

We find that MPS schedules are better when the phase shift is large and bright light is available, and LCS schedules are better when the phase shift is small and only dim light is available. The reasons are as follows. In general, schedules attempt to take the pacemaker on the shortest path to re-entrainment. For this light may be used to decrease circadian amplitude, and in doing so, is opposed by the effects of the pacemaker attempting to return amplitude to its original level (limit cycle). This is analogous to the problem of pushing a ball over a hill. The circle around the hill is like the phases of the clock along the limit cycle, and the steepness of the hill is the effect of amplitude recovery, which pushes the oscillator to the limit cycle. When light levels are too low, the amplitude recovery is stronger than the effect of light, and light cannot move the clock on most direct path between two points on the limit cycle.

Our results challenge previously held assumptions about efficient phase shifting of the human circadian clock. It has been previously suggested by many authors that a schedule that passes close to the phase singularity [22] will be sensitive to noise [7], [36], [46]. We find however that MPS schedules are in fact more robust than those that stay near the limit cycle (LCS). This is due primarily to the fact that the end-state of the optimal schedule is not at the singularity (where phase is most sensitive) but rather a point on the limit cycle (where phase is most robust). By shifting quickly, we instead predict that errors will have less time to accumulate.

We also find that the dynamics of circadian photoreception in humans has a large impact on phase shifting in minimum time. While short pulses of light can give nearly as much signal to the circadian pacemaker as continuous light [11], [12], our results agree with previous studies showing that continuous light yields a larger drive to the circadian pacemaker [21]. Moreover, in LCS there exist phase regions where light is left off in both advancing and delaying schedules. This challenges the strategy, based largely on iPRCs, that the day can be divided into exactly two regions, one where light should be presented to advance, and another where light should be presented to delay. This is due to the fact that even in LCS schedules, a small amount of amplitude suppression occurs, causing the pacemaker to leave the limit cycle until the phase shift is completed, thereby invalidating the iPRC.

Finally, we describe how schedules for complete re-entrainment in minimum time can be used to create more practical schedules for the treatment of jet lag. This is done using optimal schedules to rapidly shift CBTmin into the sleep/dark region, specifically by shifting it to the beginning of the region. This may facilitate better sleep quality in the new time zone, and may resolve many of the symptoms associated with jet lag more quickly than schedules for complete re-entrainment.

Circadian misalignment due to jetlag is a major problem for modern society. The optimal schedules presented here, perhaps especially the schedules for partial re-entrainment, bring us closer to designing schedules which may help travelers re-entrain quickly. More importantly perhaps, the principles described in this manuscript could be used to compute and design customized schedules which help individuals re-entrain while minimizing jet lag and performance lapses in practical settings, such as shift work, where many parameters such as the amount of exposure to bright light or the amount of darkness/sleep are constrained. Moreover, the method could be generalized in a straightforward way to multiple control inputs in addition to light, such as the timing of sleep, exercise, or pharmacological treatments, further accelerating re-entrainment. It could also be applied to multi-level oscillator models such as [28] or biochemical models such as [47].

We were pleased to find that the schedules we present are simple to follow, in the sense that they involve only a single daily light exposure, and that they are predicted to yield uniform results even in the presence of unpredictable factors. We found a significant effect of the circadian phototransduction system on schedules, and that some schedules match aspects of previous recommendations, e.g. avoiding morning light [13]. Considering that other, less optimal strategies are widely used, e.g. on smartphone or web applications, we hope that this methodology, and perhaps the schedules themselves, will be of significant use to circadian researchers and, eventually, to travelers and shift-workers. We also hope that methods similar to the ones presented here could be used to study other problems of optimal perturbation of biological oscillators, including those that regulate breathing or heart rhythms, or potentially to ecological or environmental problems on larger scales.

## Methods

Our methodology to compute optimal schedules consists of two major contributions. First, we define the re-entrainment problem in terms of optimal control theory. This includes computing the **isochrons** of the model. Second, we compute the optimal solution using a novel numerical algorithm based on a method originally used to optimize robotic manipulators. These steps are covered in great detail in supplemental text S1 – we summarize them briefly as follows.

The models we use [16], [17] comprise a system of ordinary differential equations. These equations should relate the state of the model, which we call 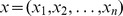, to the time 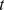 and a control (e.g. light) which we call 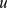 – we should be able to write them in this form:
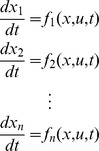
We formulate an “optimal control” problem by defining two functions of 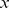 and 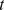: the “constraint” 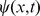 which must equal 0 at the final time 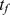, and represents the conditions we would like our solution to satisfy, and the “cost” 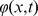, which represents the quantity we would like to minimize at the final time 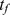.

**Figure 7:**
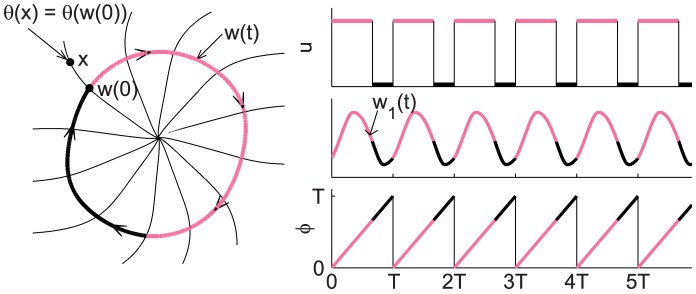
Entraining stimulus and corresponding limit cycle of the Jewett-Forger-Kronauer model. The top plot shows the entraining stimulus (LD-cycle) and the leftmost plot, the entrained limit cycle corresponding to this stimulus. Here pink corresponds to day and black to night. The middle plot shows the oscillations in the first coordinate of the entrained limit cycle. The last plot shows the phase of periodic stimulus or, equivalently, of the entrained oscillator. All significant features are labeled with the appropriate notation (See Methods, or Notation in supplemental text S1).

**Figure 8:**
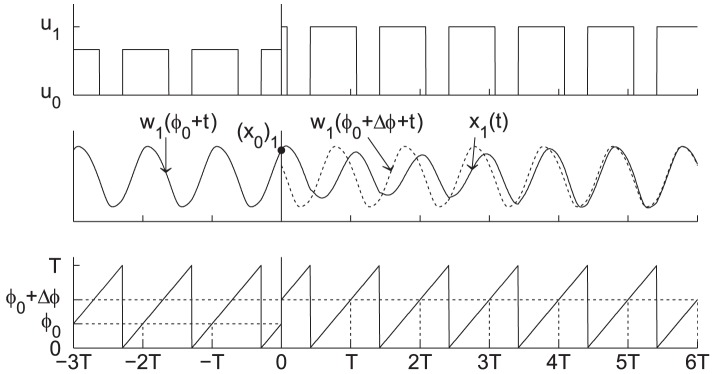
The process of re-entrainment to a phase shift of the Jewett-Forger-Kronauer Model. The top plot shows the entraining stimulus (LD-cycle) to the oscillator, plotted on a log scale, shifted by 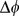 hours at time 0. The light level increases from 100 lux before time 0 to 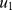 ( = 10,000 lux) after time 0. In this case 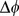 ( = 7 hours) is positive, so the schedule is advanced. The middle plot shows the oscillations in first coordinate of the model. The entrained oscillator before the phase shift is shown with a solid line, and the dotted line shows how the oscillator would behave were it entrained to the shifted stimulus. The solid line after time 0 shows the process of re-entrainment to the phase shift. The last plot shows the phase of the stimulus or, equivalently, of the entrained oscillator. Notice that the shift takes place when the phase of the stimulus (and therefore of the entrained oscillator) is 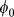 ( = 7 hours). All significant features are labeled with the appropriate notation (See Methods, or Setting up the Problem in supplemental text S1).

The constraint is defined as follows. Unlike previous works, we explicitly compute the isochrons of the model in the form of a function 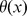, which gives the model's phase for any state 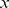. We also compute the entrained (or forced) limit-cycle 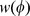, which gives the state of the entrained model as a function of the phase 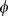, which is defined as the remainder of time 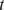 divided by 24 hours (see figure 7). We use 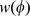 and 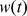 interchangeably where the meaning is clear. Beginning our optimization at phase 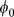 and state 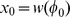, to achieve a phase shift of 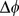 hours we require that at 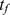 the final phase is equal to the re-entrained phase, or that 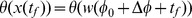 (See figure 8). Therefore, we set 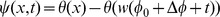. This guarantees that the final phase is exactly entrained, which has heretofore not been done.

The cost is defined in the following way. Since we would like to minimize time 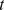, a natural cost function would be 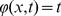. However, we would also like to control how much circadian amplitude is recovered, as more amplitude recovery is desirable. If we compute amplitude with the function 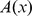, then this can be accomplished with the penalty 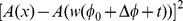, which we add to the cost with the coefficient 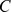: 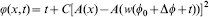. This novel approach allows us to control how much amplitude is recovered by adjusting the size of 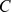.

Once we have an optimal control problem to solve, we compute its solution using a numerical algorithm. We use a novel modification of a numerical method called the Switch Time Optimization method [48]. This method assumes that the control is “bang-bang,” meaning that it switches between the minimum and maximum levels. In fact, we show that such a control is optimal (See supplemental text S1). The algorithm works by computing so-called “sensitivity functions” 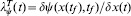 and 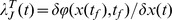, which relate changes in the state of the model at time 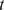 to the final constraint and final cost respectively. At each step, the algorithm takes the previous set of switching times, and using these sensitivity functions computes a set of small changes which, when added to these switching times, will decrease the cost function while keeping the constraint satisfied. The critical modification allowing this algorithm to work on the problem of minimum time re-entrainment is step 4 of the method, which precisely controls the step size. This novel contribution to the method significantly improves its rate of convergence – without it the original algorithm in [48] fails. The final algorithm is given below:

**Step 1** *Guess nominal terminal time* 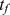 *and switching times* 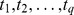 *on* 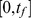 *.*
**Step 2** *Determine the trajectory* 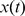 *by integrating the system equations forward from* 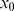 *using these switching times.*
**Step 3** *Determine the sensitivity functions* 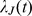 *and* 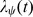 *by integrating backwards*

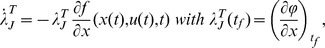
and
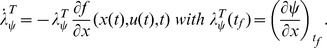

**Step 4** *Let* 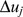 *be the jump in the control at time* 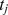 *, with positive sign for a jump “down” and negative for a jump “up.” Choose some small* 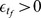 *and, denoting the fastest timescale of the problem by* 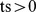 *, set*

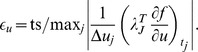

**Step 5** *Determine the optimal perturbations for decreasing* 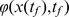


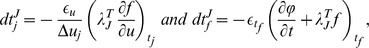

*and for increasing* 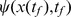


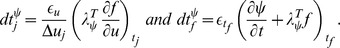

**Step 6** *Determine the effect of these perturbations on* 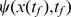


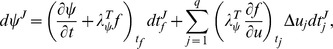

*and*

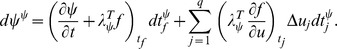

**Step 7** *Choose some small* 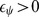 *and set*

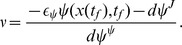

**Step 8** *Record the optimal increments* 
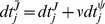
 *for* 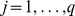 *and* 
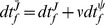
 *. Then update the solution with* 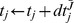 *for* 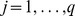 *and* 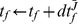 *.*

When this algorithm converges – in the sense that the guess can no longer be improved – we find that the solution satisfies a set of local optimality conditions called Pontryagin's Minimum Principle (see supplemental text S1). Hence this algorithm takes any initial guess for the control and improves it until it is locally optimal.

## Supporting Information

Figure S1 **Example of noisy light levels.** (**A**) shows the light schedule for re-entraining to a 12-hour shift in minimum time (See figure 1F). (**B**) shows this same light schedule, but with noise imitating experimentally observed variations in light intensity [40]. The random noise was produced as follows. Every 10 minutes a number was sampled from a standard normal distribution. These numbers were then linearly interpolated to get a function of time. The schedule was then transformed to a log scale and the noise was added. The result is normally distributed noise added on a log scale, with a standard deviation of one order of magnitude.
(EPS)

Figure S2 **Polar phase-amplitude resetting maps corresponding to** **figure 3** **.** Circadian phase is plotted in degrees, with 0° corresponding to entrained CBTmin (before the schedule shift occurs), while amplitude is measured on the radius. A 6 hour advance would be indicated by a shift to 90°, a 12 hour shift to 180°, and a 6 hour delay to 270°. The sleep/dark region is indicated by the shaded regions on the PARMs. The timing of entrained CBTmin in the new time zone is indicated by the dotted line. Figures (**A**)–(**O**) correspond directly to figures 3A–3O, with each subplot displaying the process re-entrainment under one of the 15 schedules considered in figure 3. The phase and amplitude of predicted CBTmin (magenta triangles) are plotted in a one-to-one correspondence with the markers of figure 3. In other words, the markers and lines of figure 3 are simply re-plotted here in phase-amplitude space. As in figure 3, re-entrainment is plotted for 4 days before the schedule shift and for 14 days after.
(EPS)

Figure S3
**Isochrons and limit cycle of the Jewett-Forger-Kronauer Model.** The limit cycle and isochrons (curves of constant phase) of the model [16] are plotted in 2-dimensional phase space [22]. The horizontal axis corresponds to the variable 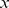 in the model; the vertical to 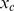. The color indicates phase by its hue and amplitude by its brightness, with white representing zero amplitude [37]. Isochrons were computed using backwards integration [42]. The white regions at (−1,1) and (1,−1) could not be computed because trajectories diverged too rapidly.(TIF)

Figure S4 **Predicted circadian phase and amplitude under the slam shift.** Days −2 and −1 correspond to a 16∶8 LD-cycle of 100 lux. At day 0 the schedule shift occurs. The brightness of light in the shifted LD-cycle (time >0) varies from 100 lux to 10,000 lux according to the labels on the subplots. Predictions were made using the Jewett-Forger-Kronauer model [16]. Days −2 and −1 may be used as a legend associating a unique hue to each phase of the oscillator. Brightness is then used to represent amplitude, with white corresponding to zero amplitude [37]. The exact coloring, based on the state of the model variables, is shown in supplemental figure S3. Figure (**A**) shows the predicted phase and amplitude under the slam shift with 10,000 lux light in the new time zone; (**B**) 1000 lux; (**C**) 500 lux; (**D**) 200 lux; (**E**) 100 lux.
(TIF)

Figure S5 **Optimal schedules for re-entrainment to 8 and 12 hour shifts of the LD-cycle (Simpler model).** Predicted core body temperature minima (CBTmin, magenta triangles) are plotted against the pattern of optimal exposure to bright light (200 lux–10,000 lux, yellow), moderate light (100 lux, white), and darkness (0 lux, black). Predicted CBTmin under noisy light levels (See supplemental figure S1), with circadian period randomly sampled from an experimentally measured distribution [18], is plotted for 20 hypothetical subjects (blue circles). The timing of entrained CBTmin in the new time zone is indicated by the dotted line. Circadian amplitude at CBTmin is indicated by the brightness of the markers, with white corresponding to zero amplitude. The subjects are initially entrained to a 16∶8 LD-cycle in moderate light. At day 0 the schedule shift occurs. Optimal schedules are grouped in rows by maximum admissible bright light level (yellow), and in columns by effected phase shift. Figures (**A**), (**B**), (**C**) use a maximum light level of 10,000 lux; (**D**), (**E**), (**F**) use 1000 lux; (**G**), (**H**), (**I**) use 500 lux; (**J**), (**K**), (**L**) use 200 lux; (**M**), (**N**), (**O**) use 100 lux. Figures (**A**), (**D**), (**G**), (**J**), (**M**) are optimal schedules for an 8-hour delay; (**B**), (**E**), (**H**), (**K**), (**N**) a 12-hour shift; (**C**), (**F**), (**I**), (**L**), (**O**) an 8-hour advance.
(EPS)

Figure S6 **Polar phase-amplitude resetting maps corresponding to supplemental figure S5 (Simpler model).** Circadian phase is plotted in degrees, with 0° corresponding to entrained CBTmin (before the schedule shift occurs), while amplitude is measured on the radius. A 6 hour advance would be indicated by a shift to 90°, a 12 hour shift to 180°, and a 6 hour delay to 270°. The sleep/dark region is indicated by the shaded regions on the PARMs. The timing of entrained CBTmin in the new time zone is indicated by the dotted line. Figures (**A**)–(**O**) correspond directly to figures S5A–S5O, with each subplot displaying the process re-entrainment under one of the 15 schedules considered in figure S5. The phase and amplitude of predicted CBTmin (magenta triangles) are plotted in a one-to-one correspondence with the markers of figure S5. In other words, the markers and lines of figure S5 are simply re-plotted here in phase-amplitude space. As in figure S5, re-entrainment is plotted for 4 days before the schedule shift and for 14 days after.
(EPS)

Figure S7 **Optimal schedules for all phase shifts (Simpler model).** This plot shows the pattern of bright light (200 lux–10,000 lux, yellow), background light (100 lux, white), and darkness (0 lux, black) under which the clock is optimally reset from the corresponding initial phase. If a vertical line is drawn on the plot, then the pattern of light and dark along this vertical line is the optimal schedule for resetting the clock from the corresponding initial phase. Figure (**A**) shows the optimal schedules for resetting the clock when 10,000 lux light is available; (**B**) 1000 lux; (**C**) 500 lux; (**D**) 200 lux; (**E**) 100 lux.
(EPS)

Figure S8 **Predicted optimal circadian phase and amplitude for all phase shifts (Simpler model).** The format is exactly the same as figure S7. Days −2 and −1 may be used as a legend associating a unique hue to each phase of the oscillator. Brightness is then used to represent amplitude, with white corresponding to zero amplitude [37]. The exact coloring, based on the state of the model variables, is shown in supplemental figure S9. Figure (**A**) shows the predicted phase and amplitude under the optimal schedules for resetting the clock when 10,000 lux light is available; (**B**) 1000 lux; (**C**) 500 lux; (**D**) 200 lux; (**E**) 100 lux.
(TIF)

Figure S9
**Isochrons and limit cycle of the Simpler model.** The limit cycle and isochrons (curves of constant phase) of the model [17] are plotted in 2-dimensional phase space [22]. The horizontal axis corresponds to the variable 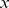 in the model; the vertical to 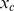. The color indicates phase by its hue and amplitude by its brightness, with white representing zero amplitude [37]. Isochrons were computed using backwards integration [42]. The white regions at (−1,−1) and (1,1) could not be computed because trajectories diverged too rapidly.(TIF)

Figure S10 **Predicted circadian phase and amplitude under the slam shift (Simpler model).** Days −2 and −1 correspond to a 16∶8 LD-cycle of 100 lux. At day 0 the schedule shift occurs. The brightness of light in the shifted LD-cycle (time >0) varies from 100 lux to 10,000 lux according to the labels on the subplots. Predictions were made using the Simpler model [17]. Days −2 and −1 may be used as a legend associating a unique hue to each phase of the oscillator. Brightness is then used to represent amplitude, with white corresponding to zero amplitude [37]. The exact coloring, based on the state of the model variables, is shown in supplemental figure S9. This format for displaying the process of re-entrainment is used in section of 1D of [22].
(TIF)

Figure S11 **Optimal one-pulse stimuli to advance or delay the clock (Simpler model).** We simulated the PRCs to all possible one-pulse stimuli for a variety of different light levels. For each light level, two stimuli were selected: the one producing the greatest advance and the one producing the greatest delay. The model was kept in total darkness before the stimulus was administered. Resulting phase shifts were measured using the concept of isochrons [22]. On the right the optimal advancing stimuli (top) and optimal delaying stimuli (bottom) are plotted for each light level. The bars indicate both the duration (bar length) and phase (midpoint) of the light stimuli relative to the timing of CBTmin (solid magenta vertical line). On the left the PRCs corresponding to each optimal stimulus length are plotted. On each PRC, the optimal phase maximizing the shift is marked by a circle (filled for advances and unfilled for delays). The PRCs corresponding to optimal advancing stimuli are drawn using solid lines, and ones corresponding to optimal delaying stimuli, using dashed lines. We found that, for low light levels and smaller shifts, the daily light exposures observed in the optimal schedules (See figures S7 and S8) matched the optimal one-pulse stimuli very closely. In particular, we find that the optimal advancing stimuli are much longer than the optimal delaying stimuli.
(EPS)

Figure S12
**Optimal trajectories corresponding to** **figure 3** **.** The optimal trajectories are plotted in phase space. The horizontal axis corresponds to the variable 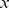 in the model; the vertical to 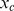. The limit cycle is shown by the dotted line and the trajectory of the model is shown by the solid line. The isochron representing entrained circadian phase at the terminal time is shown by the thick solid line. The times when the light switches on are marked by the bright purple circles; times when it switches off are marked by the dark purple xs (the control is bang-bang). The time when the optimal schedule begins is marked by a small black circle on the limit cycle. The figures (**A**)–(**O**) correspond directly to figures 3A–3O, with each subplot displaying the process re-entrainment under one of the 15 schedules considered in figure 3.(EPS)

Figure S13
**Optimal controls and Hamiltonians corresponding to** **figure 3** **.** The optimal control 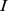 (equivalently 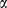 for the purposes of schedule design) is plotted in black against the quantity 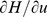, shown by a red curve. For the control to be optimal it is necessary that at each time 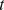, the control 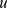 (i.e. 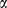) minimizes 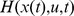 (See condition (2) in PMP2 in supplemental text S1). Since 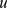 appears linearly in 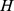, we see that 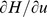 gives the coefficient of 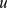 in 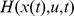. Thus when 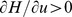 the control must take its minimum value and when 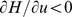 the control must take its maximum value. This is precisely what we see in all the plots on the right. Thus the condition is satisfied. The figures (**A**)–(**O**) correspond directly to figures 3A–3O, with each subplot displaying the optimal schedule and Hamiltonian derivative for one of the 15 schedules.(EPS)

Figure S14
**Optimal trajectories corresponding to figure S5 (Simpler model).** The optimal trajectories are plotted in phase space. The horizontal axis corresponds to the variable 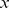 in the model; the vertical to 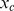. The limit cycle is shown by the dotted line and the trajectory of the model is shown by the solid line. The isochron representing entrained circadian phase at the terminal time is shown by the thick solid line. The times when the light switches on are marked by the bright purple circles; times when it switches off are marked by the dark purple xs (the control is bang-bang). The time when the optimal schedule begins is marked by a small black circle on the limit cycle. The figures (**A**)–(**O**) correspond directly to figures S5A–S5O, with each subplot displaying the process re-entrainment under one of the 15 schedules considered in figure S5.(EPS)

Figure S15
**Optimal controls and Hamiltonians corresponding to supplemental figure S5 (Simpler model).** The optimal control 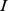 (equivalently 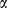 for the purposes of schedule design) is plotted in black against the quantity 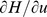, shown by a red curve. For the control to be optimal it is necessary that at each time 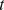, the control 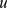 (i.e. 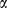) minimizes 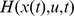 (See condition (2) in PMP2 in supplemental text S1). Since 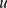 appears linearly in 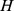, we see that 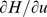 gives the coefficient of 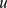 in 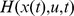. Thus when 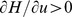 the control must take its minimum value and when 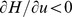 the control must take its maximum value. This is precisely what we see in all the plots on the right. Thus the condition is satisfied. The figures (**A**)–(**O**) correspond directly to figures S5A–S5O, with each subplot displaying the optimal schedule and Hamiltonian derivative for one of the 15 schedules.(EPS)

Text S1 **Detailed methods.** A detailed description of the methods is provided.
(DOC)

Text S2 **Glossary.** A glossary of terms is provided.
(DOC)
