# Supplementary figures and images for: Optimal Schedules of Light Exposure for Rapidly Correcting Circadian Misalignment
Source: PLoS Comput Biol. 2014 Apr 10;10(4):e1003523. doi: 10.1371/journal.pcbi.1003523 (PMC3983044; doi:10.1371/journal.pcbi.1003523)

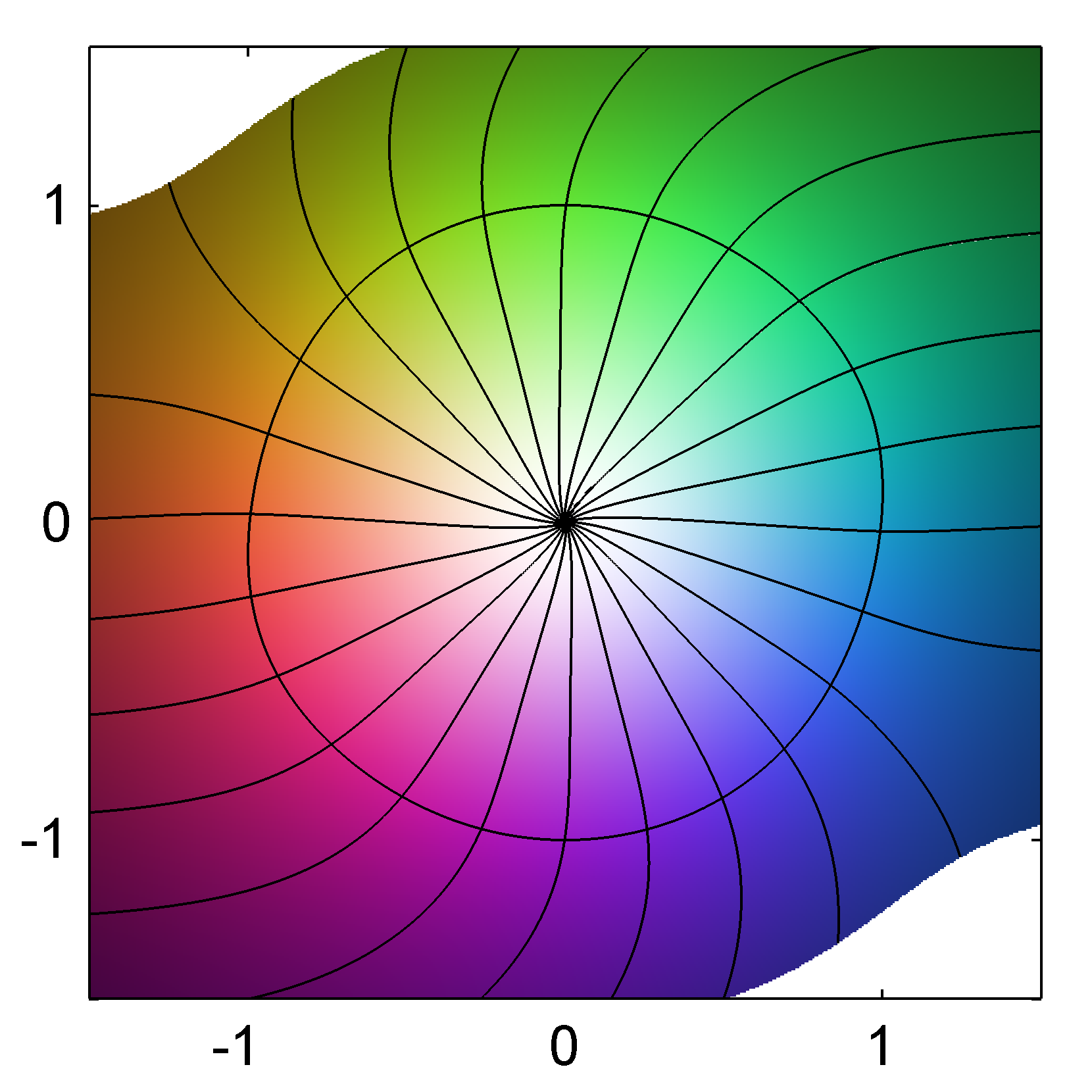

Supplement: Figure S3 — Isochrons and limit cycle of the Jewett-Forger-Kronauer Model. The limit cycle and isochrons (curves of constant phase) of the model [16] are plotted in 2-dimensional phase space [22]. The horizontal axis corresponds to the variable in the model; the vertical to . The color indicates phase by its hue and amplitude by its brightness, with white representing zero amplitude [37]. Isochrons were computed using backwards integration [42]. The white regions at (−1,1) and (1,−1) could not be computed because trajectories diverged too rapidly. (TIF) [file pcbi.1003523.s003.tif]

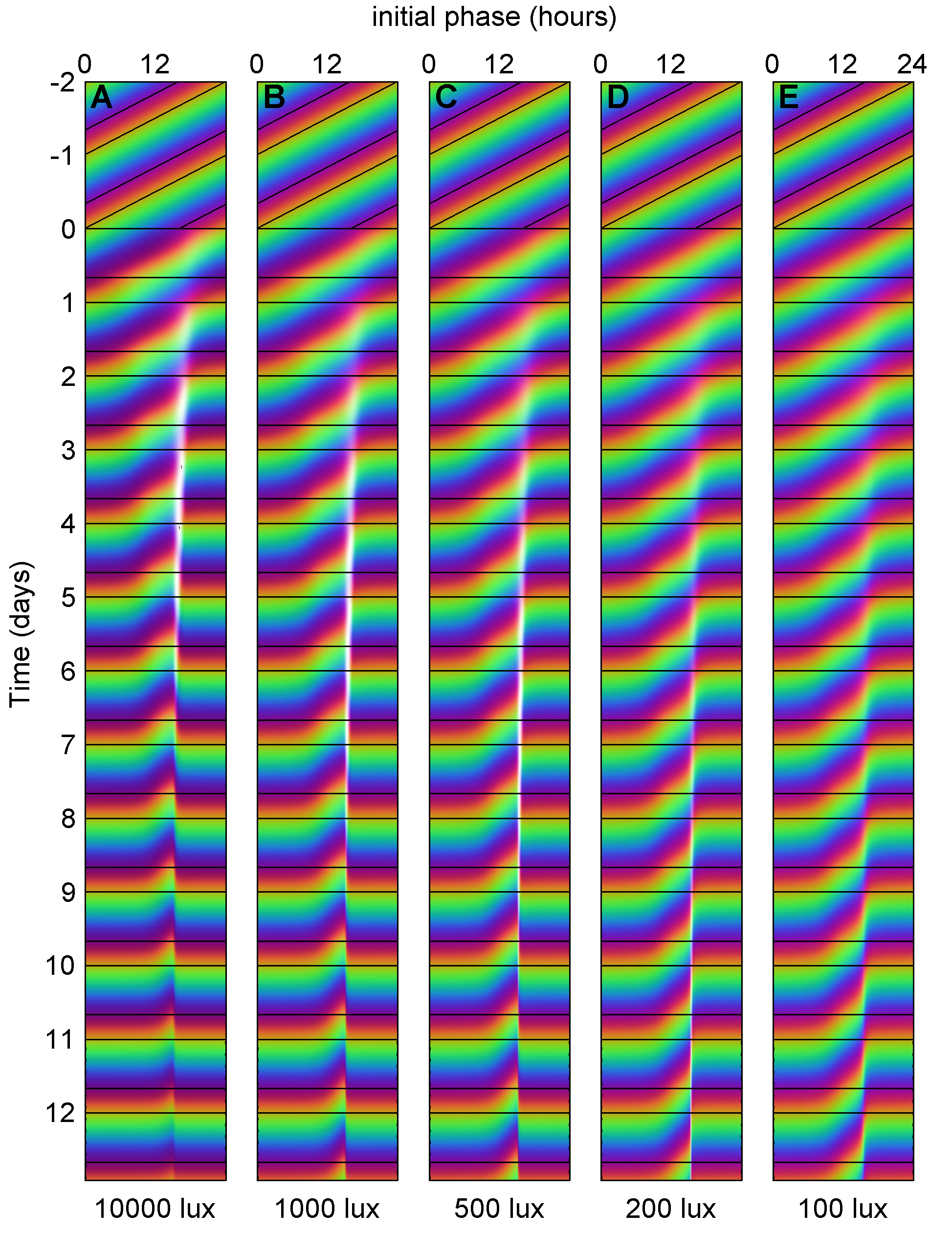

Supplement: Figure S4 — Predicted circadian phase and amplitude under the slam shift. Days −2 and −1 correspond to a 16∶8 LD-cycle of 100 lux. At day 0 the schedule shift occurs. The brightness of light in the shifted LD-cycle (time >0) varies from 100 lux to 10,000 lux according to the labels on the subplots. Predictions were made using the Jewett-Forger-Kronauer model [16]. Days −2 and −1 may be used as a legend associating a unique hue to each phase of the oscillator. Brightness is then used to represent amplitude, with white corresponding to zero amplitude [37]. The exact coloring, based on the state of the model variables, is shown in supplemental figure S3. Figure (A) shows the predicted phase and amplitude under the slam shift with 10,000 lux light in the new time zone; (B) 1000 lux; (C) 500 lux; (D) 200 lux; (E) 100 lux. (TIF) [file pcbi.1003523.s004.tif]

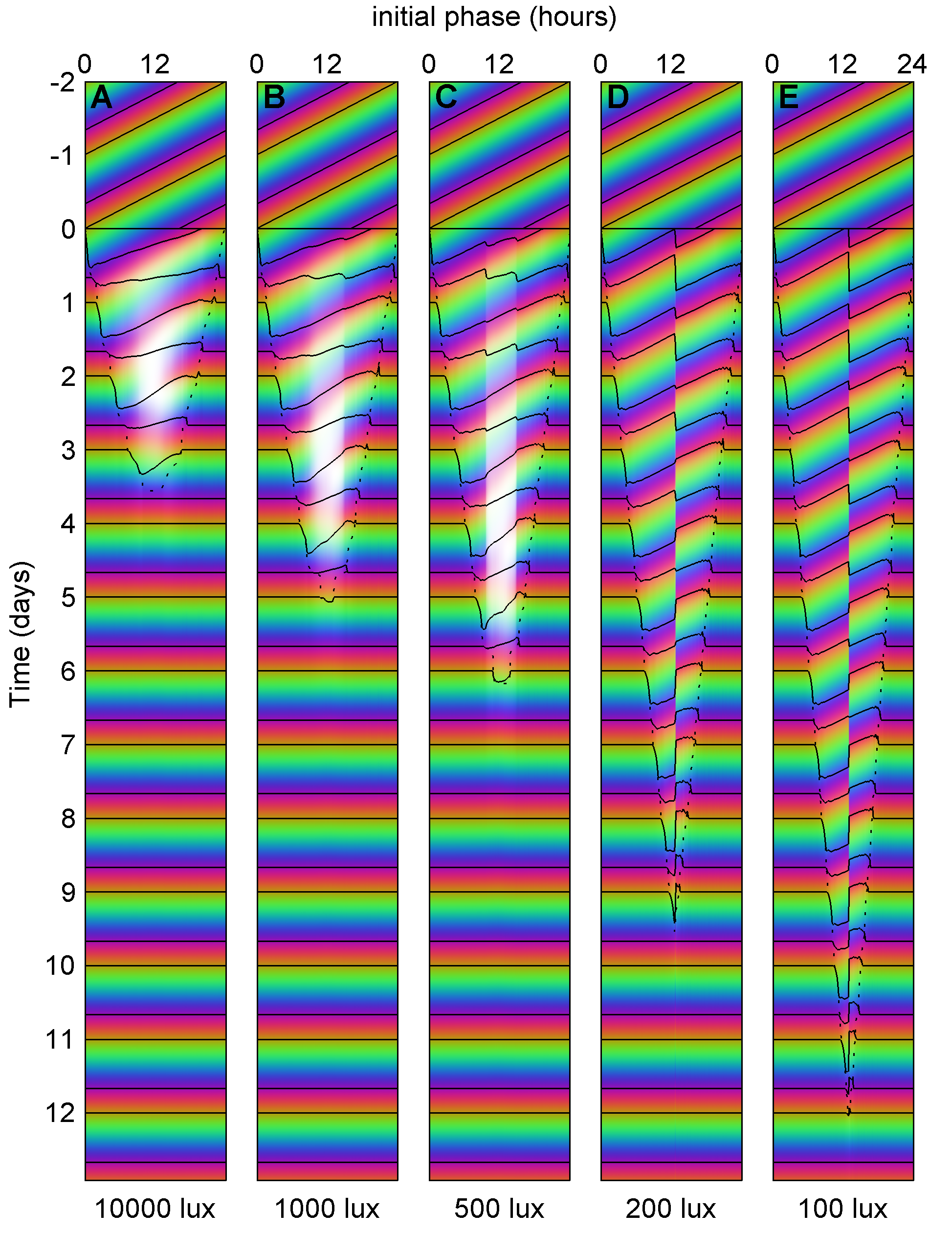

Supplement: Figure S8 — Predicted optimal circadian phase and amplitude for all phase shifts (Simpler model). The format is exactly the same as figure S7. Days −2 and −1 may be used as a legend associating a unique hue to each phase of the oscillator. Brightness is then used to represent amplitude, with white corresponding to zero amplitude [37]. The exact coloring, based on the state of the model variables, is shown in supplemental figure S9. Figure (A) shows the predicted phase and amplitude under the optimal schedules for resetting the clock when 10,000 lux light is available; (B) 1000 lux; (C) 500 lux; (D) 200 lux; (E) 100 lux. (TIF) [file pcbi.1003523.s008.tif]

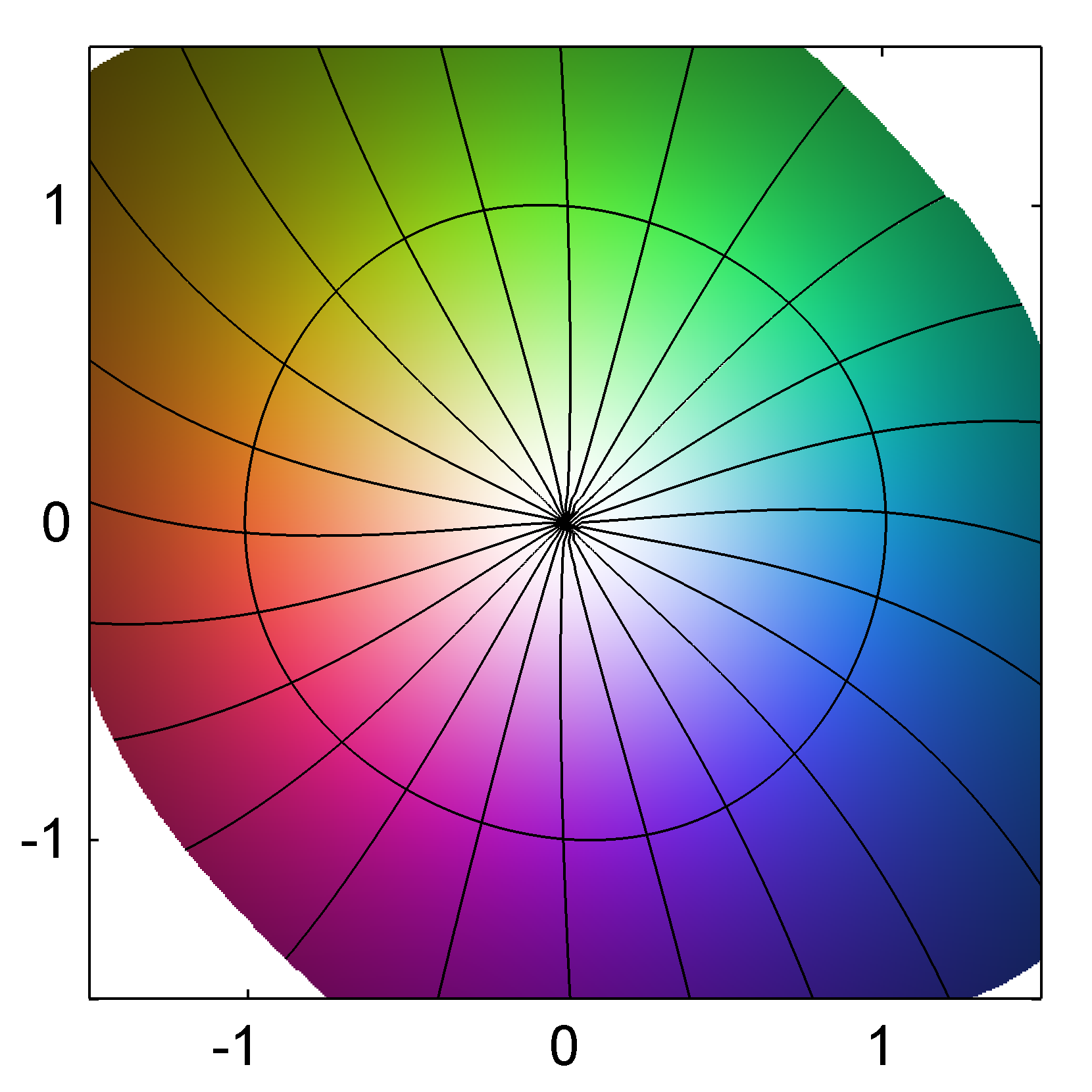

Supplement: Figure S9 — Isochrons and limit cycle of the Simpler model. The limit cycle and isochrons (curves of constant phase) of the model [17] are plotted in 2-dimensional phase space [22]. The horizontal axis corresponds to the variable in the model; the vertical to . The color indicates phase by its hue and amplitude by its brightness, with white representing zero amplitude [37]. Isochrons were computed using backwards integration [42]. The white regions at (−1,−1) and (1,1) could not be computed because trajectories diverged too rapidly. (TIF) [file pcbi.1003523.s009.tif]

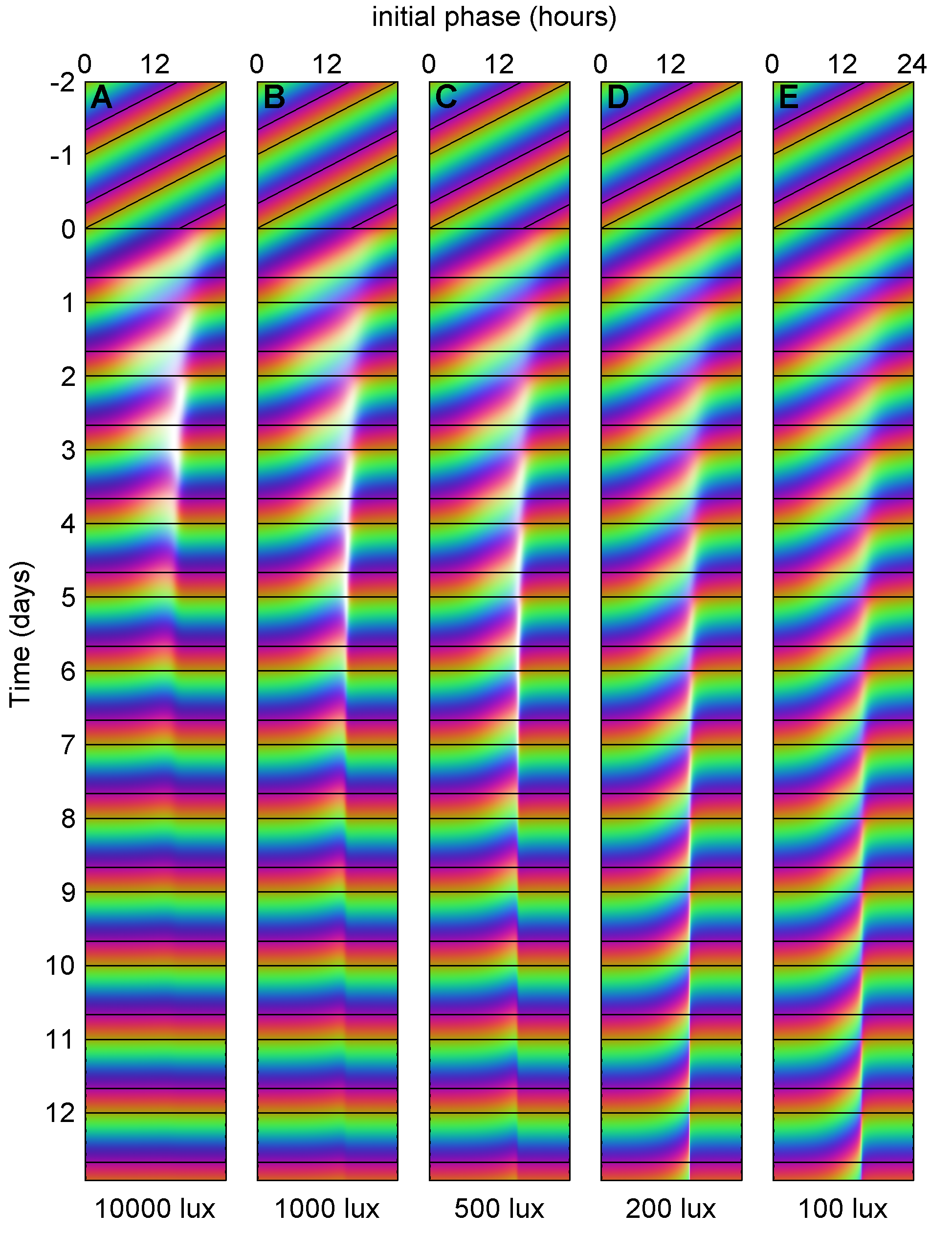

Supplement: Figure S10 — Predicted circadian phase and amplitude under the slam shift (Simpler model). Days −2 and −1 correspond to a 16∶8 LD-cycle of 100 lux. At day 0 the schedule shift occurs. The brightness of light in the shifted LD-cycle (time >0) varies from 100 lux to 10,000 lux according to the labels on the subplots. Predictions were made using the Simpler model [17]. Days −2 and −1 may be used as a legend associating a unique hue to each phase of the oscillator. Brightness is then used to represent amplitude, with white corresponding to zero amplitude [37]. The exact coloring, based on the state of the model variables, is shown in supplemental figure S9. This format for displaying the process of re-entrainment is used in section of 1D of [22]. (TIF) [file pcbi.1003523.s010.tif]
