# Supplementary material for: Optimal Schedules of Light Exposure for Rapidly Correcting Circadian Misalignment
Source: PLoS Comput Biol. 2014 Apr 10;10(4):e1003523. doi: 10.1371/journal.pcbi.1003523 (PMC3983044; doi:10.1371/journal.pcbi.1003523)
Supplement: Text S1 — Detailed methods. A detailed description of the methods is provided. (DOC) [file pcbi.1003523.s016.doc]

**Text S1: Detailed methods**

Here we describe a general method for computing the optimal schedules to re-entrain, in minimum time, a limit cycle oscillator of two or more variables. In particular, when the controlled quantity (e.g. light) appears monotonically (strictly increasing or strictly decreasing) in the model equations, we describe how this method can be modified to make it several times faster. Moreover, we discuss how the method can be adapted to highly stiff systems. Such systems appear often in biology due to the presence of widely separated timescales.

The method we present is a modification of a highly cited algorithm by Meier and Bryson called the switch time optimization (STO) method. This method is an indirect method, which means that it uses necessary conditions for an optimum to find a relatively small set of candidate solutions . The optimum is then found by picking the best one. An advantage of such a method over direct methods, which recast the problem to one that is easier to solve, is that the solutions are mathematically optimal according to Pontryagin’s Minimum Principle (PMP) .

Notation

The n-dimensional **limit cycle oscillator** (LCO) will be described by the equation for state $x=(x_{1},x_{2},\ldots,x_{n})$, external forcing $u(t)$, and time $t$, where represents $dx/dt$. The mathematical expression $\theta(x)$ will denote the **phase** of the oscillator at point $x$, defined in terms of **isochrons**. A periodic stimulus $u(t)$ to the model will have **period** $T$. Under certain conditions (see figure 13 of ) the stimulus $u(t)$ will be **entraining**, which means that the oscillator will eventually settle into **stable limit cycle** $w(t;u(\cdot))$ with period $T$ (We will write $w(t)$ instead of $w(t;u(\cdot))$ when the choice of $u$ is obvious). In other words, $w(\cdot)$ will have the property that $w(t)=w(t+T)$ for any time $t$. We thus define the phase of the entrained oscillator by (here mod means remainder). In the sequel we move back and forth between the notations $w(t)$ and where convenient. See figure 7 for a visual description of this notation.

Setting up the Problem

Suppose that the oscillator is entrained and that, at phase , the stimulus is suddenly phase shifted by . Eventually the oscillator will re-entrain: setting , we have that

.

(See figure 8).

If we can control $u(t)$ for $t\geq0$ within fixed limits $u_{0}\leq u(t)\leq u_{1}$, then we may be able to quicken re-entrainment. Specifically, we would like to solve the following problem. Find $u(t)\in[u_{0},u_{1}]$ on $[0,t_{f}]$ which controls the oscillator from to in the minimum time $t_{f}$. We call the condition the constraint.

Modifying the Constraint

Notice that the point is given by the intersection of the orbit (closed loop) $w(t)$ with the (n-1)-dimensional surface (See figure 7.) Therefore instead of requiring that

,

we can require only that

,

so long as we have a way of ensuring that $x(t_{f})$ is near $w(\cdot)$. We can do this by adding $n-1$ nonnegative, smooth penalty functions $P_{1}(x,t),P_{2}(x,t),\ldots,P_{n-1}(x,t)$ which are minimized when $x$ is near $w(\cdot)$, and $0$ when . A good example for $n=2$ (when the limit cycle is approximately a circle) is given by , where $A(\cdot)$ gives the distance from the origin, also called amplitude. (We use this penalty to optimize the circadian models.)

Our problem thus becomes the following. Find $u(t)\in[u_{0},u_{1}]$ on $[0,t_{f}]$ which controls the oscillator from to $x(t_{f})$, so that the condition is satisfied and the quantity $t_{f}+C_{1}P_{1}(x(t_{f}),t_{f})+C_{2}P_{2}(x(t_{f}),t_{f})+\cdots+C_{n-1}P_{n-1}(x(t_{f}),t_{f})$ is minimized, where$C_{1},C_{2},\ldots,C_{n-1}$ are positive constants controlling how close we want the penalty functions to be to $0$, and thus how close we want $x(t_{f})$ to be to the limit cycle.

In practical terms, we require as a constraint that the final phase be exactly entrained, but allow, by using penalties and carefully adjusting their weights, other variables (including circadian amplitude) to deviate from their entrained values within experimentally observed ranges.

Connecting the Problem to Optimal Control Theory

Letting and , it is not too hard to see that the above problem can be rewritten in the following more general form. (It is a canonical one in the field of optimal control theory.) Let $U=[u_{0},u_{1}]$. Find the control $u(t)\in U$ on $[0,t_{f}]$, allowing $t_{f}$ to vary, which transfers the system from the point $x_{0}$ to a point $x(t_{f})$ so that the condition $\psi(x(t_{f}),t_{f})=0$ is satisfied and the cost is minimized. We call the condition $\psi(x(t_{f}),t_{f})=0$ the constraint.

The method we use to find the solution works in the following way. Let $\delta u$ on $[0,t_{f}+dt_{f}]$ be an increment to the control $u$ on $[0,t_{f}]$ such that if $u$ satisfies the constraint then $u+\delta u$ does as well (when $dt_{f}>0$ the control $u$ is linearly extrapolated, see Ch. 3 of ). Since the control $u$ completely determines $J$, we can write $J[u]$. If $J[u+\delta u]\geq J[u]$ for all small increments $\delta u$ we can call the control $u$ locally optimal. Any control $u$ which is globally optimal, minimizing $J[u]$over all possible controls, must be locally optimal as well . If we are able to find the increment $\delta u$ which decreases the cost the most keeping the constraint satisfied, then given a nominal $u$ we could follow these increments to a local optimum. By trying different nominal $u$ we can find all these local optima and then simply choose the best one .

Sensitivity Functions

To motivate the method, we will first consider the case $U=(-\infty,+\infty)$. Once this is done we will return to the case $U=[u_{0},u_{1}]$. The control $u(t)$ and time $t_{f}$ determine the trajectory $x(t)$, which then determines $x(t_{f})$ and therefore the cost $J$. Thus to find the optimal increment $\delta u$ we can start by asking how a small perturbation in the states $\delta x(t)$ at a *fixed* time $t$ will effect$J$. Let’s let

$$\lambda^{T}(t)=\frac{\delta J}{\delta x(t)}$$

be the gradient of the cost with respect to a perturbation in the states at time $t$. We call $\lambda(t)$ a sensitivity function . Since , we know that

(1)

We’d like to scale this sensitivity backwards from time $t_{f}$ to earlier times $t<t_{f}$. From the Taylor expansion we know that for small $\Delta t>0$ we have (letting $f(t)$ denote $f(x(t),u(t),t)$)

$\delta x(t)=\delta x(t-\Delta t)+\frac{\partial f}{\partial x}(t-\Delta t)\delta x(t-\Delta t)\Delta t+O(\Delta t^{2})$.

Since

$$\delta J=\lambda^{T}(t)\delta x(t),$$

we can substitute to get

$$\delta J=\lambda^{T}(t)\left(\delta x(t-\Delta t)+\frac{\partial f}{\partial x}(t-\Delta t)\delta x(t-\Delta t)\Delta t\right)+O(\Delta t^{2})\text{ }$$

$=\left(\lambda^{T}(t)+\lambda^{T}(t)\frac{\partial f}{\partial x}(t-\Delta t) \Delta t\right)\delta x(t-\Delta t)+O(\Delta t^{2})$.

Since $\delta J=\lambda^{T}(t-\Delta t)\delta x(t-\Delta t)$, we have that

$\lambda^{T}(t-\Delta t)=\lambda^{T}(t)+\lambda^{T}(t)\frac{\partial f}{\partial x}(t-\Delta t)\Delta t+O(\Delta t^{2})$.

Rearranging this and taking the limit as $\Delta t\to0$, we find that

. (2)

Solving this backward from (1) gives us the sensitivity of the cost to changes in the states at any time $t$.

Suppose that, letting $h_{N}=t_{f}/N$, we can perturb the states at times $t_{n}=n h_{N}$ for $n=1,2,\ldots,N$ ($x(0)$ is fixed). Because the differential equation for $\lambda^{T}(t)$ is linear, we can superimpose the solutions to get the effect of the perturbations $\delta x(t_{n})$ on the cost $J$:

$\delta J=\sum_{n=1}^{N} \lambda^{T}(t_{n})\delta x(t_{n})$. (3)

Changes in Final Time

We are allowed to vary $t_{f}$, so we would like to see if it is possible to perturb $t_{f}$ by some amount $dt_{f}$ which would decrease the cost $J$. We have that

which, since and , is the same as

.

Thus incorporating changes in final time into (3) gives us

. (4)

Variations in the Controls

We are allowed only to change the states $x(t)$ indirectly by varying the control $u(t)$. Suppose that on the intervals $[t_{n-1},t_{n}]$ for $n=1,\ldots,N$ we are allowed to vary the control $u(t)$ by a constant $\delta u(t_{n-1})$. Then the perturbation $\delta x(t_{n})$ in the states at time $t_{n}$ resulting from a change in the control on $[t_{n-1},t_{n}]$ is given by

$\delta x(t_{n})=\frac{\partial f}{\partial u}(t_{n-1})\delta u(t_{n-1})h_{N}+O(h_{N}^{2})$.

Substituting into (4) we get

,

and taking limits as $N\to\infty$ we obtain

. (5)

In optimal control theory the expression $\lambda^{T}(t)f(x(t),u(t),t)$ is often called the Hamiltonian, and is denoted by $H(x(t),u(t),t)$. With this notation, we write

.

Notice that $(\partial H/\partial u)(t)$ can be a considered the “gradient” of $J$ with respect to the control $u(t)$ and the “gradient” with respect to $t_{f}$.

Optimal Perturbations

From (5), we see that the cost $J$ is guaranteed to decrease if $t_{f}$ is perturbed by

And the control $u(t)$ is perturbed by

(6)

for small enough values of and . If we repeatedly apply these perturbations then the cost will decrease. Since in any reasonable problem we cannot decrease the cost indefinitely, we expect that $dt_{f}^{J}$ and $\delta u^{J}(t)$ will approach $0$. This is equivalent to the statements that

and that, for each $t$,

$\frac{\partial H}{\partial u}(x(t),u(t),t)\to0$. (7)

In fact, these are the necessary conditions for optimality called Pontryagin’s Minimum Principle .

PMP1 : *If a control* $u(t)$ *and final time* $t_{f}$ *are optimal, then there exist sensitivity functions* $\lambda(t)$ *which satisfy* (1) *and* (2) *such that, letting* $H=\lambda^{T}f$*, the follow conditions are satisfied:*

*1)* *,*

*2) $H(x(t),u(t),t)=\inf_{u\in U} H(x(t),u,t)$ for each* $t$ *in* $[0,t_{f}]$.

Notice that when $U=(-\infty,+\infty)$, condition 2 implies (7). PMP1 remains true when we take $U=[u_{0},u_{1}]$ instead $U=(-\infty,+\infty)$.

Terminal Constraints

To address the issue of t­­erminal constraints, we begin by asking how changes in the controls and final time affect $\psi(x(t_{f}),t_{f})$. Replacing by $\psi$ and re-deriving equation (5) gives us

$\delta\psi=\left(\frac{\partial\psi}{\partial t}+\lambda_{\psi}^{T}f\right)_{t_{f}}dt_{f}+\int_{0}^{t_{f}} \lambda_{\psi}^{T}(t)\frac{\partial f}{\partial u}(t)\delta u(t)dt$ (8)

where

$\lambda_{\psi}^{T}(t_{f})=\left(\frac{\partial\psi}{\partial x}\right)_{t_{f}}$.

If we perturb the control and final time by $\delta u^{J}(t)$ and $dt_{f}^{J}$ then the resulting change in $\psi$, which we’ll call $d\psi^{J}$, is given by substituting $\delta u^{J}(t)$ and $dt_{f}^{J}$ into (8). We would like to somehow adjust $\delta u^{J}(t)$and $dt_{f}^{J}$ to correct for this deviation in $\psi$.

Notice that the perturbations

and

(9)

are guaranteed to *increase* $\psi$ for small enough and . Let $d\psi^{\psi}$ be the result of substituting $dt_{f}^{\psi}$ and $\delta u^{\psi}(t)$ into (8). Choosing some coefficient $\nu$, we can augment $\delta u^{J}(t)$ and $dt_{f}^{J}$ to get

$\delta u^{\bar{J}}(t)=\delta u^{J}(t)+\nu u^{\psi}(t)$,

and

$dt_{f}^{\bar{J}}=dt_{f}^{J}+\nu dt_{f}^{\psi}$,

for which the resulting change in $\psi$ will be $d\psi^{\bar{J}}=d\psi^{J}+\nu d\psi^{\psi}$.

If the nominal control $u$ satisfies the constraint $\psi=0$, then we can choose $\nu=-d\psi^{J}/d\psi^{\psi}$to make $d\psi^{\bar{J}}=0$, so that $u+\delta u^{\bar{J}}$ on $[0,t_{f}+dt_{f}^{\bar{J}}]$ also satisfies the $\psi=0$. If the nominal $u$ does not satisfy the constraint, then we can pick some small and choose . This will bring the constraint closer to 0 by .

Optimal Perturbations with Constraints

Since the equations for the sensitivity functions are linear, it’s possible to show that the augmented increments $\delta u^{\bar{J}}(t)$ and $dt_{f}^{\bar{J}}$ are actually the optimal increments associated with the augmented cost , where

,

with

, (10)

and

. (11)

If we increment the control and final time by

and

(12)

with

,

then for small enough and , the cost $J$ will decrease the most it can bringing $\psi(x(t_{f}),t_{f})$ closer to 0 by .

If we repeatedly apply these perturbations then the cost and $\psi$ will both decrease. Since in any reasonable problem we cannot decrease the cost indefinitely, we expect that $dt_{f}^{\bar{J}}$, $\delta u^{\bar{J}}(t)$, and $\psi$ will all approach $0$. Letting $H=\Lambda^{T}f$, this is equivalent to the statements that

,

$\frac{\partial H}{\partial u}(x(t),u(t),t)\to0$ (13)

for each $t$, and

$\psi(x(t_{f}),t_{f})\to0$.

In fact, these are the necessary conditions for optimality when terminal constraints are present, called Pontryagin’s Minimum Principle with terminal constraints .

PMP2 : *If a control* $u(t)$ *and final time* $t_{f}$ *are optimal, then there exist a constant* $\nu$ *and sensitivity functions* $\Lambda(t)$ *satisfying* (10) *and* (11) *such that, letting* $H=\Lambda^{T}f$*, the following conditions are satisfied:*

*1)* ,

*2) $H(x(t),u(t),t)=\inf_{u\in U} H(x(t),u,t)$ for each* $t$ *in* $[0,t_{f}]$,

*3)* $\psi(x(t_{f}),t_{f})=0$.

Notice that when $U=(-\infty,+\infty)$, condition 2 implies (13). PMP2, like PMP1, remains true when we take $U=[u_{0},u_{1}]$ instead $U=(-\infty,+\infty)$.

Bang-bang Controls

Suppose that for each $x$ and $t\geq0$, $f(x,u,t)$ is a monotonic function of $u$. Let $U=[u_{0},u_{1}]$ instead of $U=(-\infty,+\infty)$ as in the previous sections. From PMP2, we have that an optimal control $u(t)$ must, at each time $t$, minimize$H(x(t),u,t)$. But since $f(x,u,t)$ is a monotonic function of $u$ we have that $H(x(t),u,t)=\lambda^{T}(t)f(x(t),u,t)$ is minimized on the boundary of $U$. Thus the optimal control must be, at any moment in time, either $u_{0}$ or $u_{1}$. This kind of control, that switches between its maximum and minimum admissible values, is called “bang-bang”.

If we consider only bang-bang controls then all the information over the interval $[0,t_{f}]$ is contained in the times $t_{1},t_{2},\ldots,t_{q}$ at which the control switches from maximum to minimum or from minimum to maximum. An increment $\delta u(\cdot)$ of the control can therefore be treated as change in its switching times $dt_{j}$ for $j=1,\ldots,q$. Thus an integral of $\delta u(\cdot)$ is a sum

$\int_{0}^{t_{f}} \delta u(t)dt=\sum_{j=1}^{q} \Delta u_{j}dt_{j}$,

where $\Delta u_{j}=u(t_{j}-)-u(t_{j}+)$, where $(\cdot)-$ means the limit from the left and $(\cdot)+$ means the limit from the right.

Hence (5) and (8) become

and

$$d\psi=\left(\frac{\partial\psi}{\partial t}+\lambda_{\psi}^{T}f\right)_{t_{f}}dt_{f}+\sum_{j=1}^{q} \left(\lambda_{\psi}^{T}\frac{\partial f}{\partial u}\right)_{t_{j}}\Delta u_{j}dt_{j}$$

so (6) and (9) become

,

and

.

Stiff Problems

Problems in biology are often stiff in the sense that both very fast and very slow timescales are present. If the problem is stiff, then often the increments $dt_{j}^{J}$tend to become very large, violating the linearity assumption (Our conclusions are based on first-order approximations.) One possible solution is to make very small, but often it must be made so tiny that the problem cannot be solved in a reasonable amount of time. However, when the problem is bang-bang and the fastest timescale of the problem is known, another solution is available. At each iteration, we can choose so that $\max_{j}\left|dt_{j}^{J}\right|$ matches the timescale of the problem, which we denote by “ts,” by setting

.

In this way we can take, at each step, the largest increment which does not violate the linearity assumption. This is the critical modification which allows us to optimize and , which are both quite stiff.

The Switch Time Optimization Algorithm (STO)

Incorporating the above changes for bang-bang controls and summarizing the method we described, we arrive at the following modification of the algorithm proposed by Meier and Bryson in .

**Step 1** *Guess nominal terminal time $t_{f}$ and switching times* $t_{1},t_{2},\ldots,t_{q}$ *on* $[0,t_{f}]$*.*

**Step 2** *Determine the trajectory* $x(t)$ *by integrating the system equations forward from* $x_{0}$ *using these switching times.*

**Step 3** *Determine the sensitivity functions* $\lambda_{J}(t)$ *and* $\lambda_{\psi}(t)$ *by integrating backwards*

*with* ,

and

*with* $\lambda_{\psi}^{T}(t_{f})=\left(\frac{\partial\psi}{\partial x}\right)_{t_{f}}$.

**Step 4**  *Let $\Delta u_{j}$ be the jump in the control at time $t_{j}$, with positive sign for a jump “down” and negative for a jump “up.”* *Choose some small*  *and, denoting the fastest timescale of the problem by* $\text{ts}>0$*, set*

.

**Step 5** *Determine the optimal perturbations for decreasing*

and ,

*and for increasing* $\psi(x(t_{f}),t_{f})$

and .

**Step 6** *Determine the effect of these perturbations on* $\psi(x(t_{f}),t_{f})$

$d\psi^{J}=\left(\frac{\partial\psi}{\partial t}+\lambda_{\psi}^{T}f\right)_{t_{f}}dt_{f}^{J}+\sum_{j=1}^{q} \left(\lambda_{\psi}^{T}\frac{\partial f}{\partial u}\right)_{t_{j}}\Delta u_{j}dt_{j}^{J}$,

*and*

$d\psi^{\psi}=\left(\frac{\partial\psi}{\partial t}+\lambda_{\psi}^{T}f\right)_{t_{f}}dt_{f}^{\psi}+\sum_{j=1}^{q} \left(\lambda_{\psi}^{T}\frac{\partial f}{\partial u}\right)_{t_{j}}\Delta u_{j}dt_{j}^{\psi}$.

**Step 7** *Choose some small*  *and set*

.

**Step 8** *Record the optimal increments* $dt_{j}^{\bar{J}}=dt_{j}^{J}+\nu dt_{j}^{\psi}$ *for* $j=1,\ldots,q$ *and* $dt_{f}^{\bar{J}}=dt_{f}^{J}+\nu dt_{f}^{\psi}$*. Then update the solution with* $t_{j}\leftarrow t_{j}+dt_{j}^{\bar{J}}$ *for* $j=1,\ldots,q$ *and* $t_{f}\leftarrow t_{f}+dt_{f}^{\bar{J}}$*.*

Once the cost stops decreasing and the constraint is satisfied, we check that the solutions given by this algorithm satisfy the necessary conditions of Pontryagin’s Minimum Principle (PMP2).

The Jewett-Forger-Kronauer Model

The Jewett-Forger-Kronauer model of the human circadian system is given by the following system of differential equations:

$\frac{\partial x}{\partial t}=\frac{\pi}{12}\left(x_{c}+\mu\left(\frac{1}{3}x+\frac{4}{3}x^{3}-\frac{256}{105}x^{7}\right)+B\right)$,

$$\frac{\partial x_{c}}{\partial t}=\frac{\pi}{12}\left(qBx_{c}-x\left[\left(\frac{24}{0.99729\tau_{x}}\right)^{2}+kB\right]\right)$$

$\frac{\partial n}{\partial t}=60\left(\alpha(I)(1-n)-\beta n\right)$,

where

$\alpha(I)=\alpha_{0}\left(\frac{I}{I_{0}}\right)^{p}$,

and

$B=G\alpha(I)(1-n)(1-0.4x)(1-0.4x_{c})$.

with $\alpha_{0}=0.05$, $\beta=0.0075$, $G=33.75$, $I_{0}=9500$, $p=0.5$, $k=0.55$, $\mu=0.13$, $q=1/3$, and $\tau_{x}=24.2$.

The variable $x$ is assigned to closely reflect the endogenous core body temperature, while $x_{c}$ is an associated complementary variable. The phase of the oscillator is defined relative to the timing of the minimum of $x$, called $x_{\text{min}}$, and is related to the timing of CBTmin by the formula

,

where hour.

$n$ models how light effects the circadian system (phototransduction), by processing the light input $I$ into a term $B$ which directly influences the oscillator. Notice that $I$ only enters into the model through $\alpha(I)$ and that $\alpha(\cdot)$ is monotonic (and thus invertible). We can therefore forget about $I$ temporarily and consider $\alpha$ as the input to the model.

Notice as well that when $\alpha=0$, the value of $n$ does not influence $x$ and $x_{c}$ at all. Also, when $\alpha=0$, $n$ approaches $0$ as $t$ increases. Thus when $\alpha=0$ we can simply set $n=0$ and treat the model as a 2-dimensional oscillator. Therefore, while the oscillator is 3-dimensional, the dynamics of the unforced oscillator are essentially 2-dimensional. The isochrons of the model, projected onto the $x,x_{c}$-plane, are shown in supplemental figure S3.

The Simpler Model

The Simpler model of the human circadian system is given by the following system of differential equations:

$\frac{\partial x}{\partial t}=\frac{\pi}{12}\left(x_{c}+B\right)$,

$\frac{\partial x_{c}}{\partial t}=\frac{\pi}{12}\left(\mu\left(x_{c}-\frac{4}{3}x_{c}^{3}\right)-x\left[\left(\frac{24}{0.99669\tau_{x}}\right)^{2}+kB\right]\right)$,

$\frac{\partial n}{\partial t}=60\left(\alpha(I)(1-n)-\beta n\right)$,

where

$\alpha(I)=\alpha_{0}\left(\frac{I}{I_{0}}\right)^{p}$,

and

$B=G\alpha(I)(1-n)(1-0.4x)(1-0.4x_{c})$.

with $\alpha_{0}=0.05$, $\beta=0.0075$, $G=33.75$, $I_{0}=9500$, $p=0.5$, $k=0.55$, $\mu=0.23$, and $\tau_{x}=24.2$. The roles of $x$, $x_{c}$, and $n$ are the same as for the Jewett-Forger-Kronauer model, except with hours. The isochrons of the model, projected onto the $x,x_{c}$-plane, are shown in supplemental figure S9.

Computing the Isochrons

The isochrones were computed using the method given by Izhikevich (See Ch. 10 ex. 3 of ) for 2-dimensional LCOs (we set $n=0$), with some minor modifications. The initial isochron segment was computed using Malkin’s method (See Ch. 10 ex. 12 of ), instead of taking a radial line segment. Also, instead of using Euler’s method for the backward integrations we used the leap-frog method , which works better for oscillatory solutions.

Optimizing the Schedules

Each model is a 3-dimensional LCO which behaves like a 2-dimensional LCO. We proceed just as in the 3-dimensional case, except instead of using two penalty function (See Setting up the Problem) we only need to use one. We set

,

where

$$A(\mathbf{x})=A(x,x_{c},n)=\sqrt{x^{2}+x_{c}^{2}}$$

is the circadian amplitude (See and ). We set

$C_{1}=200$,

which ends up recovering approximately 85-95% of amplitude (with the exception of figure 1G, in which we used $C_{1}=10$).

We choose a 16:8 LD-cycle of 100 lux as our entraining stimulus ($T=24$ hours). Without loss of generality we then find, for each maximum light level (100 lux to 10,000 lux) and each between 0 and 24, the optimal schedule to re-entrain the oscillator to a shift of . Hence we find the optimal schedules to reset the oscillator from any initial phase (See figures 4 and 5 and supplemental figures S7 and S8).

Since $\alpha$ appears linearly in both models, the optimal control is always bang-bang. Thus we use the STO algorithm to compute the optimal schedules. We use the shifted LD-cycle in the new time zone as our initial guess for $u$. We do this because the LD-cycle is entraining, so for a large enough final time ($t_{f}\approx200-300$) we are guaranteed that the constraint is nearly satisfied. This is very desirable for the initial guess.

The fastest timescale of the model is on the order of 10 minutes . Thus we set “ts” to 0.1 (6 min) (See The Switch Time Optimization Algorithm). When $t_{f}$ is large and $x(t_{f})$ is close to the limit cycle, we find that . Thus we set (6 min) to match the timescale of the problem. It can be made larger but this increases the chance of violating the linearity assumptions.

We then fix the number of iterations, making it large enough so that the control can settle at a local optimum. Estimating this number is not too difficult. For example, when , 500 iterations will decrease $t_{f}$ by approximately 50 hours. When , 500 iterations will decrease $t_{f}$ by approximately 100 hours. Once we obtain a solution, we verify that it satisfies conditions (1)-(3) of PMP2.

The optimal trajectories corresponding to figure 3 are plotted in phase-space in supplemental figure S12, demonstrating visually that $x(t_{f})$ is on the desired isochron and condition (3) of PMP2 is satisfied. The derivative of the Hamiltonian, $\partial H/\partial u$, is plotted against the control in supplemental figure S13, demonstrating visually that condition (2) of PMP2 is satisfied as well. These plots are also shown for the Simpler model (supplemental figures S14 and S15), corresponding to supplemental figure S5. Condition (1) is easily checked by seeing if $dt_{f}\approx0$, with $dt_{f}$ alternating between extremely small positive and negative values.

Designing schedules for partial re-entrainment

It has been suggested that symptoms associated with jet lag and shiftwork could be alleviated if the much weaker condition that CBTmin fall within the sleep/dark (SD) region is met . In particular, some have claimed that it is desirable for CBTmin to occur at the beginning of the SD region. This has been found to facilitate sleep, since it feels good to fall asleep at CBTmin. In fact, free-running humans in temporal isolation fall asleep at CBTmin and sleep a normal amount of time.

For these reasons, a widely accepted rule of thumb for resolving jet lag is to place CBTmin at the start of the SD region as rapidly as possible, with the understanding that symptoms may begin to abate when CBTmin enters the SD region. Thus instead of finding schedules which cause complete circadian re-entrainment in minimum time, a reasonable alternative is to find schedules which place CBTmin at the start of the SD region in minimum time.

Fortunately, the latter problem can be framed in terms of the former. Consider a 24-hour LD cycle with $L$ hours of light followed by $D$ hours of darkness, and let $\Phi$ denote the **phase angle of entrainment**. When the light level is 100 lux, $L=16$, and $D=8$, we find that $\Phi=21.58$ (according to the Jewett-Forger-Kronauer model ). In other words, CBTmin occurs 2.42 hours before the end of the dark period. Suppose is the desired phase shift and is the phase at which the schedule shift occurs (See Methods, especially figure 8). Notice that $\Phi-L$ gives the timing of CBTmin relative to the start of the dark region (e.g. $\Phi-L=1$ hour means that CBTmin occurs 1 hour after start of the dark region). In our example $\Phi-L=5.58$ hours.

If we were to complete a shift of hours, then CBTmin would occur $\Phi-L$ hours past dusk in the new time zone. In other words, we would overshoot dusk by $\Phi-L$ hours. To correct this, we can simply advance (increase) by this amount. Thus, completely re-entraining to a shift of hours would place CBTmin at dusk. For instance, in figure 1G, we have hours (a 12 hour delay), and hours.

Another important consideration, besides phase, is circadian amplitude. It has been observed, as a general principle of circadian clocks, that greater circadian amplitude corresponds to better circadian adaptation . When we compute optimal schedules, we are able to adjust, using a parameter $C_{1}$, how much amplitude the schedules must recover. A large value of $C_{1}$ severely penalizes amplitude suppression at the end of the schedule (See Modifying the Constraint in supplemental text S1); a small value does not.

The choice of $C_{1}$ governs the tradeoff between time to re-entrainment and amplitude recovery. The smaller $C_{1}$ is, the faster re-entrainment occurs, at the cost of weaker restrictions on final amplitude (it may be large or small). In most of the schedules presented here, $C_{1}=200$, recovering 85-95% of final amplitude. When the goal is to resolve jet lag however, it may be beneficial to emphasize shifting CBTmin into the proper region as quickly as possible. To demonstrate this, we set $C_{1}=10$ in figure 1G, placing almost no importance on the final amplitude.

Finally, we note that figure 4, by summarizing optimal schedules for complete re-entrainment to every possible shift, can be used to effect partial re-entrainment as well. For example, if hours, simply locate hours (equivalently $24-6.42=17.58$ hours) on figure 4 and draw a vertical line downwards. The corresponding schedule will be an optimal schedule for partial re-entrainment.

References and Notes:

1. Meier EB, Bryson AE (1990) Efficient algorithm for time-optimal control of a two-link manipulator. Journal of Guidance, Control, and Dynamics 13: 859-866.

2. Bryson JAE, Ho Y-C (1975) Applied Optimal Control: Optimization, Estimation and Control: Taylor & Francis.

3. Subchan DS, Zbikowski R (2009) Computational Optimal Control: Tools and Practice: Wiley.

4. Pontryagin LS, G. BV, V. GR (1962) The Mathematical Theory of Optimal Processes: Wiley-Interscience.

5. Kronauer RE, Forger DB, Jewett ME (1999) Quantifying human circadian pacemaker response to brief, extended, and repeated light stimuli over the phototopic range. Journal of biological rhythms 14: 501.

6. Gelfand IM, Fomin SV (2000) Calculus of Variations (Dover Books on Mathematics): Dover Publications.

7. Jewett ME, Forger DB, Kronauer RE (1999) Revised limit cycle oscillator model of human circadian pacemaker. Journal of Biological Rhythms 14: 493-499.

8. Forger DB, Jewett ME, Kronauer RE (1999) A Simpler Model of the Human Circadian Pacemaker. Journal of Biological Rhythms 14: 533-538.

9. Izhikevich EM (2010) Dynamical Systems in Neuroscience: The Geometry of Excitability and Bursting (Computational Neuroscience): The MIT Press.

10. Dahlquist G, Bjorck A, Mathematics (2003) Numerical Methods (Dover Books on Mathematics): Dover Publications.

11. Eastman CI, Burgess HJ (2009) How to travel the world without jet lag. Sleep medicine clinics 4: 241.

12. Winfree AT (2001) The geometry of biological time: Springer Verlag.
