# Supplementary material for: Optimal Schedules of Light Exposure for Rapidly Correcting Circadian Misalignment
Source: PLoS Comput Biol. 2014 Apr 10;10(4):e1003523. doi: 10.1371/journal.pcbi.1003523 (PMC3983044; doi:10.1371/journal.pcbi.1003523)
Supplement: Text S2 — Glossary. A glossary of terms is provided. (DOC) [file pcbi.1003523.s017.doc]

**Text S2. Glossary**

**bang-bang.** A control which switches abruptly between two states. Usually these states correspond to upper and lower bounds on the values a control is permitted to take. See 1§5 of .

**circadian amplitude.** Describes the intensity of the circadian rhythm, and is often indicated by the difference between the peak and the trough of an oscillating measurable, such as CBT.

**entraining stimulus.** A periodic stimulus which will eventually force the oscillator into a rhythm with the same frequency as the stimulus and with a specific phase relationship to the stimulus (called the phase angle of entrainment). For example, an LD cycle will cause the circadian clock to settle into a 24-hour rhythm, with CBTmin (the phase of the oscillator) occurring approximately 3 hours before dawn (the phase of the stimulus). See 10.3 of .

**instantaneous phase response curve.** A phase response curve to a stimulus of infinitesimally small duration. If the phase response scales linearly with the duration of the stimulus, an iPRC can be used to derive the PRC to a stimulus of any duration. Typically the phase of the stimulus is measured on the horizontal axis and the resulting change in phase *per unit stimulus duration* on the vertical axis.

**isochron.** A way to define the phase of a stable limit cycle oscillator away from the limit cycle. An isochron is a curve (or surface) such that the trajectories of any two points on the isochron will eventually converge. Such points are said to have the same phase. See 10.1.2 of .

**limit cycle.** An isolated closed trajectory. See Ch 7 of .

**limit cycle oscillator.** A system which has a limit cycle. If the limit cycle is stable, then a system which exhibits self-sustained oscillations. See Ch 7 of .

**period.** The time taken for an oscillation to occur.

**phase.** A variable used to describe the state of an oscillating system. If a marker event is chosen (such as the spiking of a neuron or the minimum of core body temperature) then phase is defined to be the time since the last event. See 10.1.1 of .

**phase angle of entrainment.** The relationship between the timing of an oscillator and the timing of the entraining stimulus.

**phase response curve.** A curve illustrating the relationship between the phase at which some stimulus (e.g. a light pulse) is administered and the resulting change in phase. Typically the phase of the stimulus is measured on the horizontal axis and the resulting change in phase on the vertical axis.

**slam shift.** An abrupt shift in the LD cycle corresponding to instantaneous travel between time zones.

References and Notes:

1. Pontryagin LS, G. BV, V. GR (1962) The Mathematical Theory of Optimal Processes: Wiley-Interscience.

2. Izhikevich EM (2010) Dynamical Systems in Neuroscience: The Geometry of Excitability and Bursting (Computational Neuroscience): The MIT Press.

3. Strogatz SH (1994) Nonlinear dynamics and chaos: With applications to physics, biology, chemistry, and engineering: Westview Pr.
